# The Right Key, the Wrong Lock: TIGIT Checkpoint Blockade and the Road to Precision Immunotherapy

**DOI:** 10.3390/pharmaceutics18080970

**Published:** 2026-08-07

**Authors:** Shukur Wasman Smail, Hawro Taha Hamza, Mohammed Awat Ali, Raya Kh. Yashooa, Wissam Albeer Nooh, Ahmed Abdulrazzaq Bapir, Dlzar B. Rahman, Mohammed O. Rahman, Hiba A. Haseeb, Nivar B. Maaruf, Shadyar O. Majeed, Iman Ezzat, Christer Janson

**Affiliations:** 1 College of Pharmacy, Cihan University-Erbil, Erbil 44001, Kurdistan Region, Iraq; 2 Department of Biology, College of Science, Salahaddin University-Erbil, Erbil 44001, Kurdistan Region, Iraq; shadiarumar@gmail.com; 3 Department of Medical Science, Respiratory Medicine, and Allergology, Uppsala University and University Hospital, 75185 Uppsala, Sweden; 4 Department of Oncology, Nanakali Teaching Hospital, Erbil 44001, Kurdistan Region, Iraq; hawrotaha@gmail.com; 5 Faculty of General Medicine, Koya University, Koya 44023, Kurdistan Region, Iraq; mohammedawat751@gmail.com; 6 Department of Biology, College of Education for Pure Sciences, University of Al-Hamdaniya, Mosul 41002, Iraq; raya.yashooa@uohamdaniya.edu.iq (R.K.Y.);; 7 Department of Medical Laboratory Technology, Erbil Health and Medical Technical College, Erbil Polytechnic University, Erbil 44001, Kurdistan Region, Iraq; ahmed.mhm20@epu.edu.iq; 8 Department of Genetics Unit, Bio Diagnostic Center (BDC), Erbil 44001, Kurdistan Region, Iraqmohammedomer8535@gmail.com (M.O.R.); hbaarjumand@gmail.com (H.A.H.); nivarbotan@gmail.com (N.B.M.); 9 Department of Biomedical Sciences, Creighton University School of Medicine, Omaha, NE 68178, USA; imanezzat@creighton.edu

**Keywords:** TIGIT, CD155, CD226, immune checkpoint blockade, precision immunotherapy, tumor microenvironment, PD-1, biomarker-guided therapy, T-cell exhaustion, cancer immunotherapy

## Abstract

T-cell immunoreceptor with immunoglobulin and immunoreceptor tyrosine-based inhibitory motif (ITIM) domains (TIGIT) emerged as one of the most promising next-generation immune checkpoint targets following the success of programmed cell death protein 1 (PD-1), programmed death-ligand 1 (PD-L1), and cytotoxic T-lymphocyte-associated protein 4 (CTLA-4) blockade. TIGIT suppresses antitumor immunity through interaction with cluster of differentiation 155 (CD155), inhibition of CD226-mediated co-stimulation, and promotion of immunosuppressive regulatory T-cell (Treg) activity within the tumor microenvironment (TME). Strong preclinical evidence demonstrated that TIGIT blockade, particularly in combination with PD-1/PD-L1 inhibition, restored T-cell and natural killer (NK) cell function and produced durable antitumor responses in multiple tumor models, leading to rapid clinical development. Despite this compelling biological rationale, most late-stage clinical programs failed to reproduce early success. Although the phase II CITYSCAPE trial showed encouraging activity in PD-L1-high non-small cell lung cancer (NSCLC), subsequent phase III trials, including SKYSCRAPER-01, SKYSCRAPER-02, SKYSCRAPER-03, SKYSCRAPER-14, AdvanTIG-302, KEYVIBE, and STAR-221, failed to improve survival outcomes or meet primary endpoints. The notable exception was SKYSCRAPER-08 in esophageal squamous cell carcinoma, suggesting that TIGIT blockade may be effective only in selected biological contexts. This review critically examines the molecular biology of the TIGIT–CD155–CD226 axis, its role in immune regulation and tumor immune evasion, and the preclinical and clinical evidence supporting TIGIT-targeted therapy. Particular emphasis is placed on understanding the causes of clinical failure, including CD226 loss during T-cell exhaustion, checkpoint network redundancy, Fc-engineering uncertainty, immunosuppressive TMEs, inadequate biomarker-guided patient selection, and tumor-type-specific dependence on the TIGIT pathway. We also present original bioinformatics analyses demonstrating that broader checkpoint network signatures outperform *TIGIT* expression alone for patient stratification. Finally, we evaluate emerging solutions including biomarker-guided precision immunotherapy, Fc-optimized antibodies, bispecific checkpoint inhibitors, TIGIT-engineered chimeric antigen receptor T-cell (CAR-T) cells, radiotherapy combinations, and multi-checkpoint blockade. Collectively, current evidence suggests that the future of TIGIT-directed therapy lies not in universal checkpoint inhibition but in biologically informed, precision-guided immunotherapy strategies.

## 1. Introduction

Immune checkpoint inhibitors (ICIs) targeting programmed cell death protein-1 (PD-1), programmed death-ligand 1 (PD-L1), and cytotoxic T-lymphocyte-associated protein 4 (CTLA-4) have transformed cancer therapy, producing durable clinical responses across multiple malignancies. Despite this success, only a subset of patients derives long-term benefit, while primary and acquired resistance remain major barriers to effective treatment, underscoring the need for additional immune checkpoint targets and more effective combination strategies [1,2,3].

Among the emerging checkpoint molecules, T-cell immunoreceptor with immunoglobulin and immunoreceptor tyrosine-based inhibitory motif (ITIM) domains (TIGIT) has gained considerable attention as a next-generation immunotherapeutic target. TIGIT is expressed on activated cluster of differentiation 4^+^ (CD4^+^) and CD8^+^ T cells, natural killer (NK) cells, and regulatory T cells (Tregs) and functions primarily through interaction with the CD155 (poliovirus receptor (PVR)), which is frequently overexpressed on tumor cells and tumor-associated myeloid cells [4,5,6]. Upon ligand engagement, TIGIT suppresses antitumor immunity via direct inhibitory signaling, competition with the co-stimulatory receptor CD226 (DNAX accessory molecule-1 (DNAM-1)), and promotion of tolerogenic dendritic cells (DCs) responses [7,8,9]. Consequently, the TIGIT–CD226–CD155 axis functions as an immune regulatory system analogous to the CTLA-4/CD28 pathway, in which inhibitory signaling predominates over immune activation [10].

Within the tumor microenvironment (TME), TIGIT plays a central role in tumor immune evasion and lymphocyte exhaustion. Elevated TIGIT expression is commonly observed on tumor-infiltrating CD8^+^ T cells and NK cells, where it frequently coexists with other inhibitory receptors such as PD-1, T-cell immunoglobulin and mucin-domain containing-3 (TIM-3), and lymphocyte activation gene-3 (LAG-3) reflecting a state of profound functional exhaustion characterized by diminished cytokine production and impaired cytotoxicity [11,12,13]. In parallel, TIGIT is highly enriched on intratumoral Tregs, where it enhances suppressive activity and promotes the production of immunoregulatory cytokines including interleukin-10 (IL-10) and transforming growth factor-β (TGF-β) [14,15]. This dual activity, simultaneous inhibition of effector immune cells and enhancement of regulatory mechanisms, has made TIGIT a particularly attractive target for cancer immunotherapy.

The therapeutic rationale for TIGIT blockade was strongly supported by preclinical studies. Genetic deletion of TIGIT enhanced antitumor immunity and improved tumor control in several murine cancer models without causing significant autoimmunity. Furthermore, antibody-mediated TIGIT blockade restored T-cell and NK-cell function, with the greatest efficacy observed when combined with PD-1/PD-L1 inhibition [16,17,18]. Mechanistic studies subsequently demonstrated that TIGIT and PD-1 converge on the suppression of CD226 signaling through distinct pathways, providing a strong biological basis for dual checkpoint blockade [19].

These encouraging preclinical findings stimulated rapid clinical development, resulting in more than 70 registered clinical trials evaluating anti-TIGIT antibodies such as tiragolumab, vibostolimab, domvanalimab, and ociperlimab, predominantly in combination with PD-1/PD-L1 inhibitors [20]. Early clinical enthusiasm was driven by the phase II CITYSCAPE trial, in which tiragolumab combined with atezolizumab improved response rates and progression-free survival in PD-L1-high non-small-cell lung cancer (NSCLC). However, subsequent phase III studies—including SKYSCRAPER-01, SKYSCRAPER-02, SKYSCRAPER-03, and SKYSCRAPER-14—failed to meet their primary endpoints, and several other development programs were terminated following futility analyses [21,22,23,24,25]. Notably, the phase III SKYSCRAPER-08 trial in esophageal squamous cell carcinoma (ESCC) was the only major study to demonstrate significant improvements in both progression-free and overall survival (OS) [26].

These setbacks have shifted attention from TIGIT as a universal checkpoint target toward a more nuanced understanding of its biology. Emerging evidence indicates that factors such as CD226 downregulation, checkpoint redundancy, Fc-dependent antibody effects, immunosuppressive TMEs, and the absence of predictive biomarkers may limit therapeutic efficacy. Consequently, current research focuses on precision immunotherapy approaches incorporating biomarker-guided patient selection, optimized antibody engineering, bispecific antibodies, chimeric antigen receptor T-cell (CAR-T) integration, and rational combination therapies. Understanding why TIGIT therapies failed clinically may ultimately be as important as understanding why they initially appeared so promising.

## 2. Molecular and Functional Overview of TIGIT

TIGIT is an inhibitory immune checkpoint receptor encoded by a gene located on chromosome 3q13.31. The human TIGIT gene consists of five exons and encodes a transmembrane protein of approximately 244 amino acids. Structurally, TIGIT contains a single extracellular immunoglobulin variable (IgV) domain, a transmembrane region, and a cytoplasmic tail harboring an ITIM and an immunoglobulin tail tyrosine (ITT)-like motif, which are essential for inhibitory signaling. TIGIT is predominantly expressed in lymphoid tissues and is conserved across species, indicating an important role in immune regulation [4,7,27].

**Figure 1:**
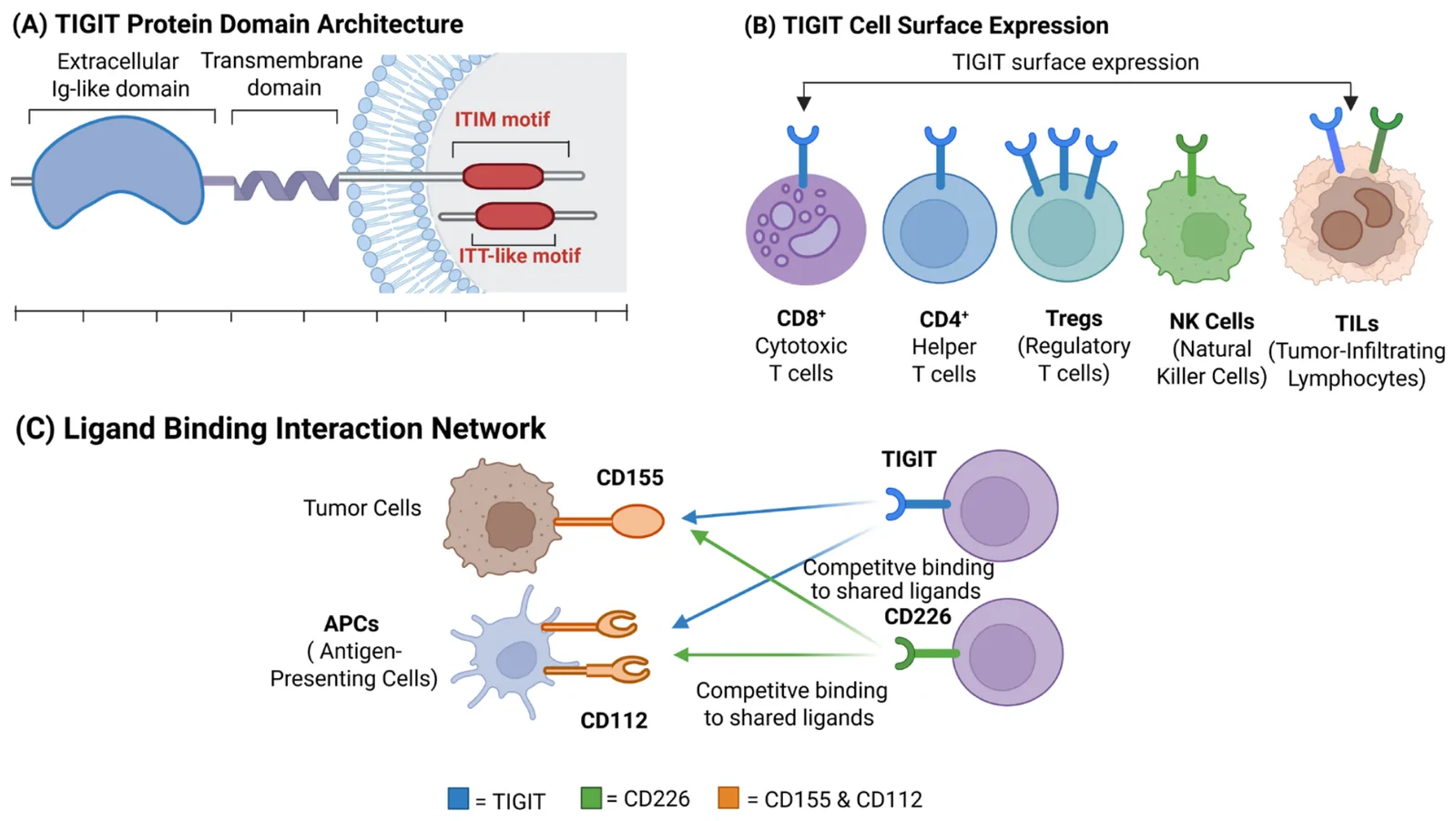
TIGIT Structure, Expression, and Ligand Interactions. Three-panel figure illustrating (**A**) the domain architecture of the TIGIT protein (extracellular Ig-like domain, transmembrane domain, intracellular ITIM and ITT-like motifs); (**B**) its expression across CD8^+^ T cells, CD4^+^ T cells, Tregs, NK cells, and TILs; and (**C**) its ligand binding network, showing how TIGIT outcompetes the co-stimulatory receptor CD226 for CD155 and CD112, converting a pro-activation signal into immune suppression. TIGIT; ITIM, immunoreceptor tyrosine-based inhibitory motif; ITT, immunoglobulin tail tyrosine; Tregs, regulatory T cells; NK cells, natural killer cells; TILs, tumor-infiltrating lymphocytes; APCs, antigen-presenting cells; TME, tumor microenvironment; CD, cluster of differentiation. Created in BioRender. Smail, S. (2026) https://BioRender.com/c4ng21a.

TIGIT is expressed on activated CD4^+^ T cells, CD8^+^ T cells, Tregs, and NK cells (Figure 1). Expression increases following lymphocyte activation and is particularly elevated in chronically stimulated or exhausted T cells during chronic infection and cancer. TIGIT-expressing Tregs exhibit enhanced suppressive activity, while TIGIT signaling in NK cells reduces degranulation and cytotoxicity. High TIGIT expression on tumor-infiltrating lymphocytes is commonly associated with an immunosuppressed phenotype and impaired antitumor immunity [4,5,11,16].

The principal ligands of TIGIT are CD155, CD112, and CD113 (Table 1). Among these ligands, CD155 is the dominant and highest-affinity binding partner and is widely expressed on antigen-presenting cells, tumor cells, macrophages, DCs, and several non-hematopoietic tissues. CD112 also participates in TIGIT-mediated inhibition, although with lower affinity than CD155, whereas CD113 interacts more weakly and its functional significance remains less well characterized. Overexpression of CD155 and CD112 has been reported in multiple malignancies, contributing to tumor immune evasion. TIGIT competes with the activating receptor CD226 for binding to these shared ligands, particularly CD155, thereby shifting immune responses toward suppression [4,5,11,16,28].

**Figure 2:**
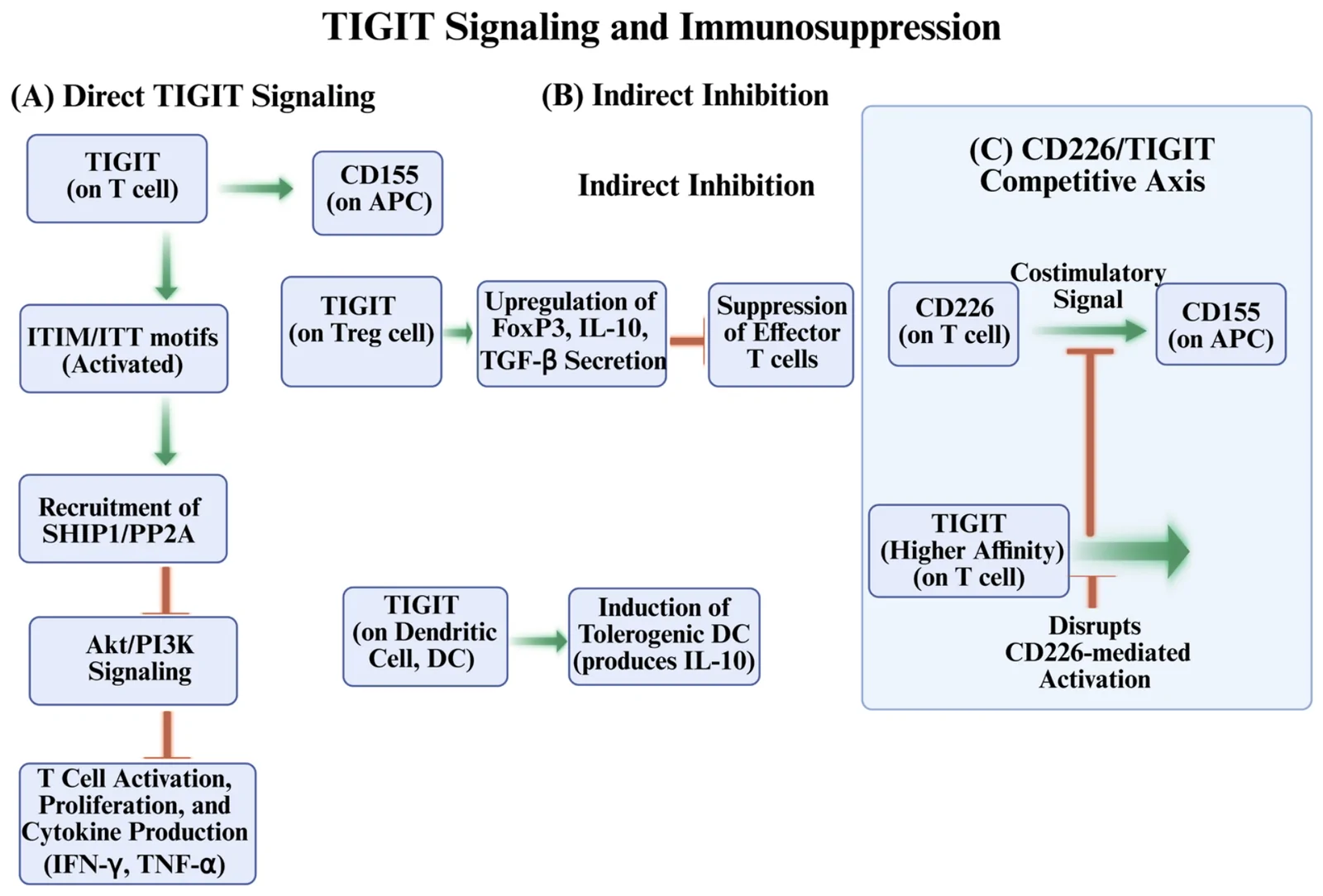
TIGIT Signaling Pathway and Immunosuppression Mechanisms. Three-panel figure covering (**A**) direct intracellular signaling via ITIM/ITT phosphorylation → SHIP1/PP2A recruitment → Akt/PI3K inactivation → suppression of T cell proliferation and cytokine production (IFN-γ, TNF-α, IL-2); (**B**) indirect suppression through Forkhead Box P3 (FOXP3+) Treg stabilization (IL-10, TGF-β) and tolerogenic DC induction; and (**C**) the CD226/TIGIT competitive axis and how CD226 downregulation in exhausted TILs creates a feed-forward suppression loop in the TME. TIGIT; APCs, antigen-presenting cells; ITIM, immunoreceptor tyrosine-based inhibitory motif; ITT, immunoglobulin tail tyrosine; SHIP1, Src homology 2-containing inositol phosphatase 1; PP2A, protein phosphatase 2A; PI3K, phosphoinositide 3-kinase; IFN-γ, interferon-gamma; TNF-α, tumor necrosis factor-alpha; FoxP3, forkhead box P3; IL-10, TGF-β, transforming growth factor-beta; DCs, dendritic cells. Green arrows indicate activation/stimulatory signaling; orange T-bar symbols indicate inhibitory signaling. Created in BioRender. Smail, S. (2026) https://BioRender.com/b4fanvf.

Upon ligand engagement, phosphorylation of the ITT-like motif recruits growth factor receptor-bound protein 2 (Grb2) and Src homology 2 domain-containing inositol 5-phosphatase 1 (SHIP1), initiating inhibitory signaling that suppresses phosphoinositide 3-kinase/protein kinase B (PI3K/Akt)-dependent activation pathways (Figure 2). Consequently, TIGIT inhibits proliferation, cytokine production, granule polarization, and cytotoxic activity of T cells and NK cells. In addition to direct intracellular inhibition, TIGIT promotes immunosuppression indirectly through interaction with CD155 on antigen-presenting cells, inducing tolerogenic responses characterized by increased IL-10 production and reduced co-stimulatory activity. Through these mechanisms, TIGIT acts as a key regulator of immune homeostasis and contributes to immune dysfunction within the tumor microenvironment [16,19,36].

## 3. Role of TIGIT in the Tumor Microenvironment

TIGIT is a key inhibitory checkpoint that contributes to immune dysfunction within the TME. Increased TIGIT expression on tumor-infiltrating CD8^+^ T cells, CD4^+^ T cells, Tregs, and NK cells is associated with impaired antitumor immunity and facilitates tumor immune escape. Engagement of TIGIT by CD155 suppresses effector-cell activation, resulting in reduced proliferation, diminished cytotoxicity, defective immune-synapse formation, and decreased production of interferon-gamma (IFN-γ) and tumor necrosis factor-alpha (TNF-α), and IL-2. These effects promote the development of an exhausted phenotype that is commonly observed in both solid tumors and hematological malignancies [16,37,38].

A prominent feature of TIGIT-mediated immunosuppression is its association with Tregs. TIGIT^+^ Tregs represent a highly suppressive population enriched within tumors and contribute substantially to local immune tolerance. These cells enhance suppression of effector lymphocytes and support the establishment of tolerogenic antigen-presenting cells (APCs), leading to increased production of IL-10 and TGF-β and reduced antitumor immune activity. Accumulation of TIGIT^+^ Tregs has been correlated with poor prognosis and resistance to immune checkpoint therapy in several cancer types [14,39,40].

TIGIT is frequently co-expressed with PD-1, TIM-3, and LAG-3 on exhausted lymphocytes. Among tumor-infiltrating immune cells, TIGIT^+^PD-1^+^ populations exhibit the greatest degree of dysfunction, characterized by reduced proliferation, impaired cytokine secretion, and weakened cytotoxic responses. The cooperative activity of these checkpoints sustains immune exhaustion and limits effective tumor control. Consequently, simultaneous blockade of TIGIT and PD-1 has emerged as a promising therapeutic strategy for restoring antitumor immunity [11,13,19].

TIGIT expression varies across cancer types but is generally elevated in immune-infiltrated malignancies. High expression has been reported in melanoma, NSCLC, colorectal cancer (CRC), pancreatic cancer, breast cancer, acute myeloid leukemia (AML), chronic lymphocytic leukemia (CLL), and multiple myeloma (Table 2). Across these tumors, elevated TIGIT expression is consistently associated with enhanced immune suppression, persistence of exhausted immune cell populations, and disease progression. Continuous expression of CD155 within the TME further reinforces inhibitory signaling and contributes to maintenance of an immunosuppressive environment [41,42,43,44,45].

## 4. Original Bioinformatics Validation of the TIGIT: CD226 Axis

To empirically test whether TIGIT expression alone is sufficient to describe the co-inhibitory/co-stimulatory balance relevant to immunotherapy response, as discussed in Section 3, we performed three complementary original analyses using publicly available transcriptomic datasets.

### 4.1. Pan-Cancer Expression and Survival Analysis

**Figure 3:**
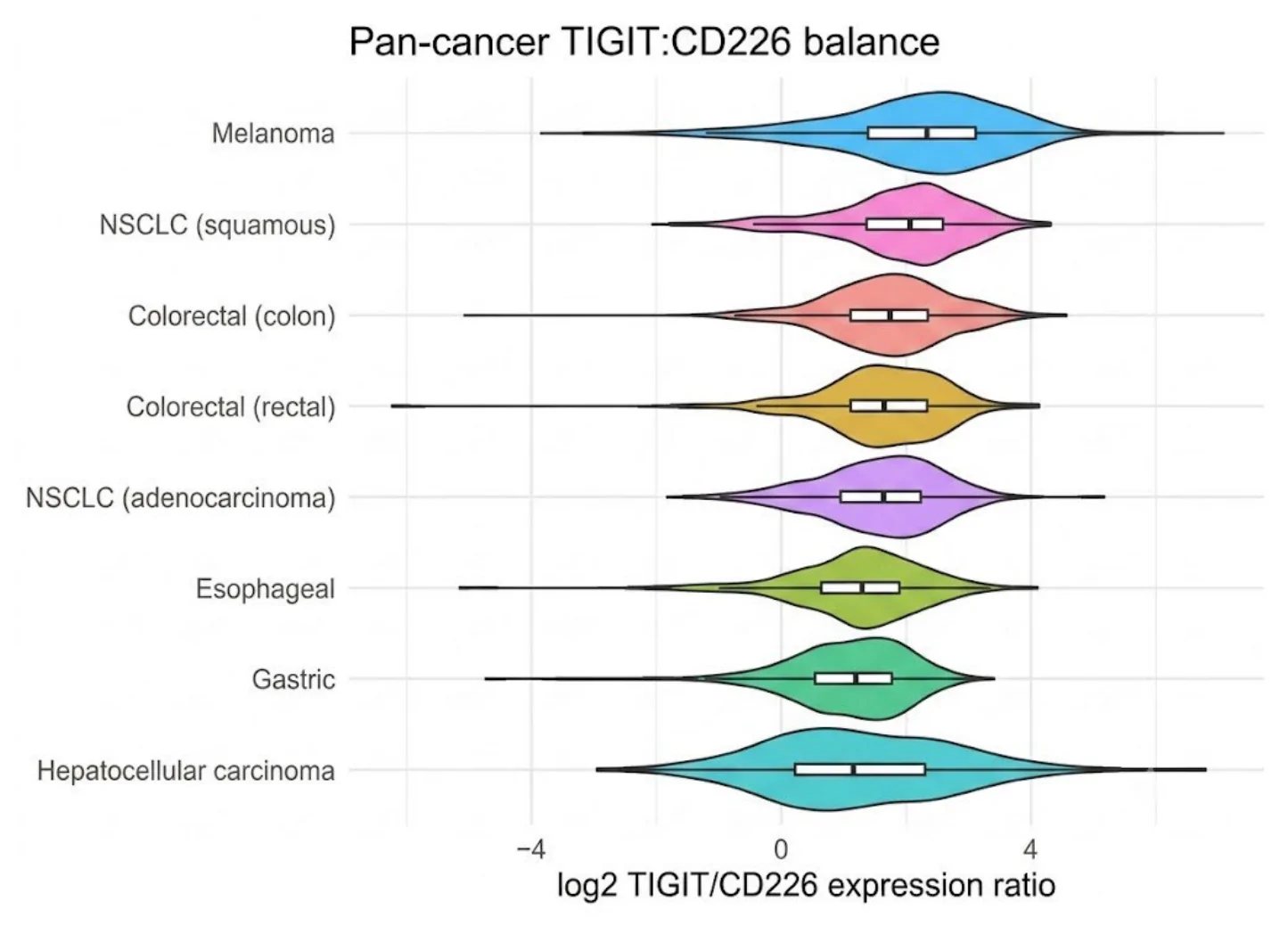
Pan-cancer distribution of the log_2_ *TIGIT*:*CD226* expression ratio across eight cancer types (TCGA Pan-Cancer Atlas, *n* = 3336 samples). Violin plots show the full distribution; embedded boxplots show median and interquartile range. A ratio > 0 indicates *TIGIT* dominance over *CD226*. *TIGIT*, *T-cell immunoreceptor with Ig* and *ITIM domains*; *CD226*, *cluster of differentiation 226* (*DNAX Accessory Molecule-1*, *DNAM-1*); *NSCLC*, *non-small cell lung cancer*; IQR, Interquartile Range; and log_2_, base-2 logarithm.

Batch-effect-corrected RNA-sequencing data, expressed on a log_2_-transformed scale, for eight cancer types corresponding to those emphasized elsewhere in this review, lung adenocarcinoma (LUAD), lung squamous cell carcinoma (LUSC), skin cutaneous melanoma (SKCM), colon adenocarcinoma (COAD), rectum adenocarcinoma (READ), liver hepatocellular carcinoma (LIHC), stomach adenocarcinoma (STAD), and esophageal carcinoma (ESCA), were obtained from The Cancer Genome Atlas (TCGA) Pan-Cancer Atlas via the UCSC Xena platform (https://xenabrowser.net) [55]. For each of 3336 tumor samples, we quantified expression of TIGIT, CD226, and five additional checkpoint genes (*programmed cell death protein* (*PDCD1*), *LAG3*, *Hepatitis A virus cellular receptor 2* (*HAVCR2*), *CD28*, and *inducible T-cell costimulator* (*ICOS*)) (Figure 3). A composite co-inhibitory score (mean z-score of *TIGIT*, *PDCD1*, *LAG3*, and *HAVCR2*) and co-stimulatory score (mean z-score of *CD226*, *CD28*, and *ICOS*) were computed per sample, together with a simple log_2_ TIGIT:CD226 expression ratio. Overall survival association was assessed per cancer type using univariate Cox proportional hazards regression (*n* = 3235 samples with complete survival annotation).

**Figure 4:**
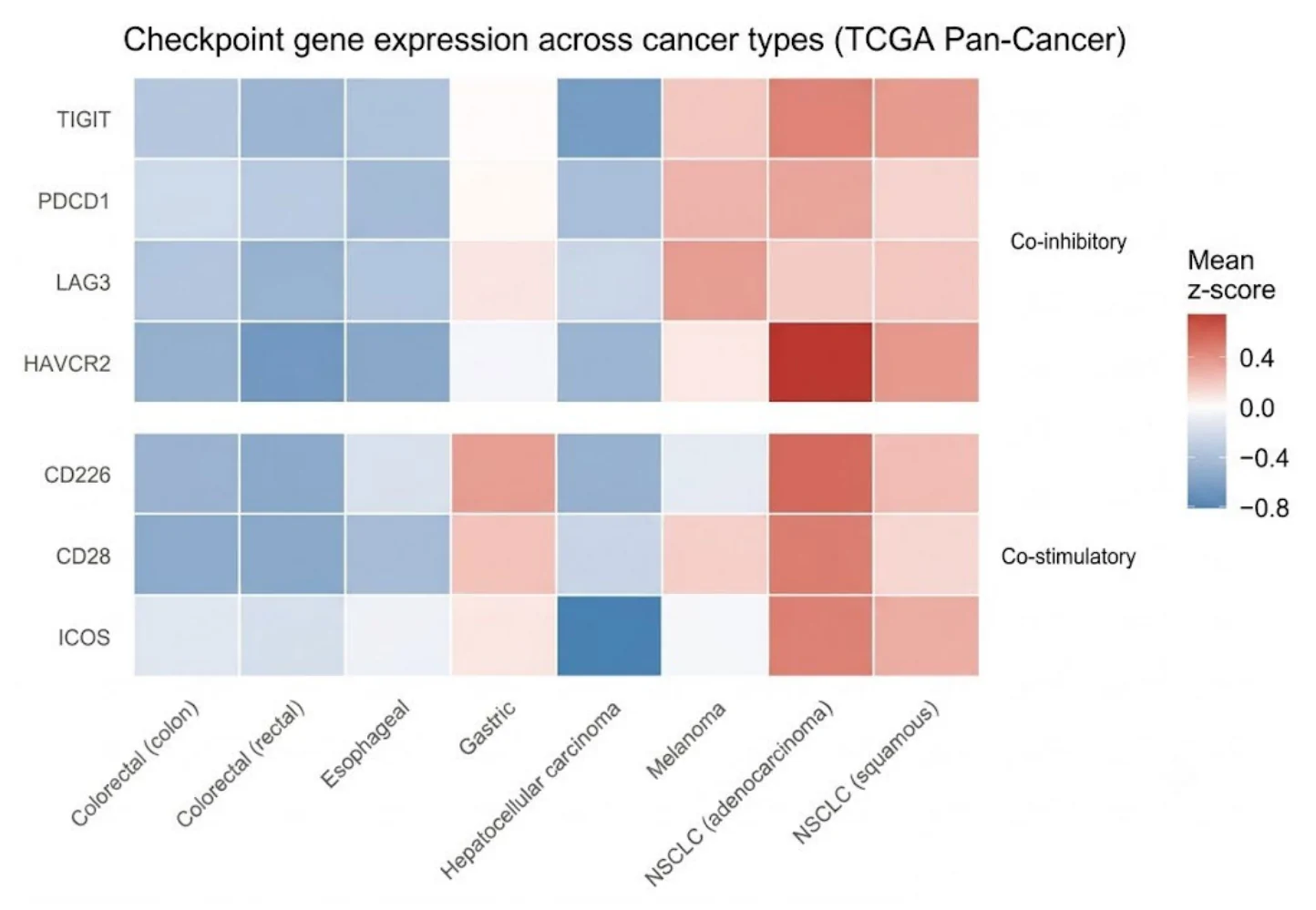
Heatmap of mean z-score expression for seven checkpoint genes (co-inhibitory: *TIGIT*, *PDCD1*, *LAG3*, and *HAVCR2*; co-stimulatory: *CD226*, *CD28*, and *ICOS*) across the same eight TCGA cancer types. *TIGIT*, T-cell immunoreceptor with Ig and ITIM domains; *PDCD1*, *programmed cell death 1*; *LAG3*, *lymphocyte activation gene 3*; *HAVCR2*, *hepatitis A virus cellular receptor 2*; *CD226*, *cluster of differentiation 226* (*DNAM-1*, *DNAX accessory molecule-1*); *CD28*, *cluster of differentiation 28*; and *ICOS*, *inducible T-cell costimulator*.

**Figure 5:**
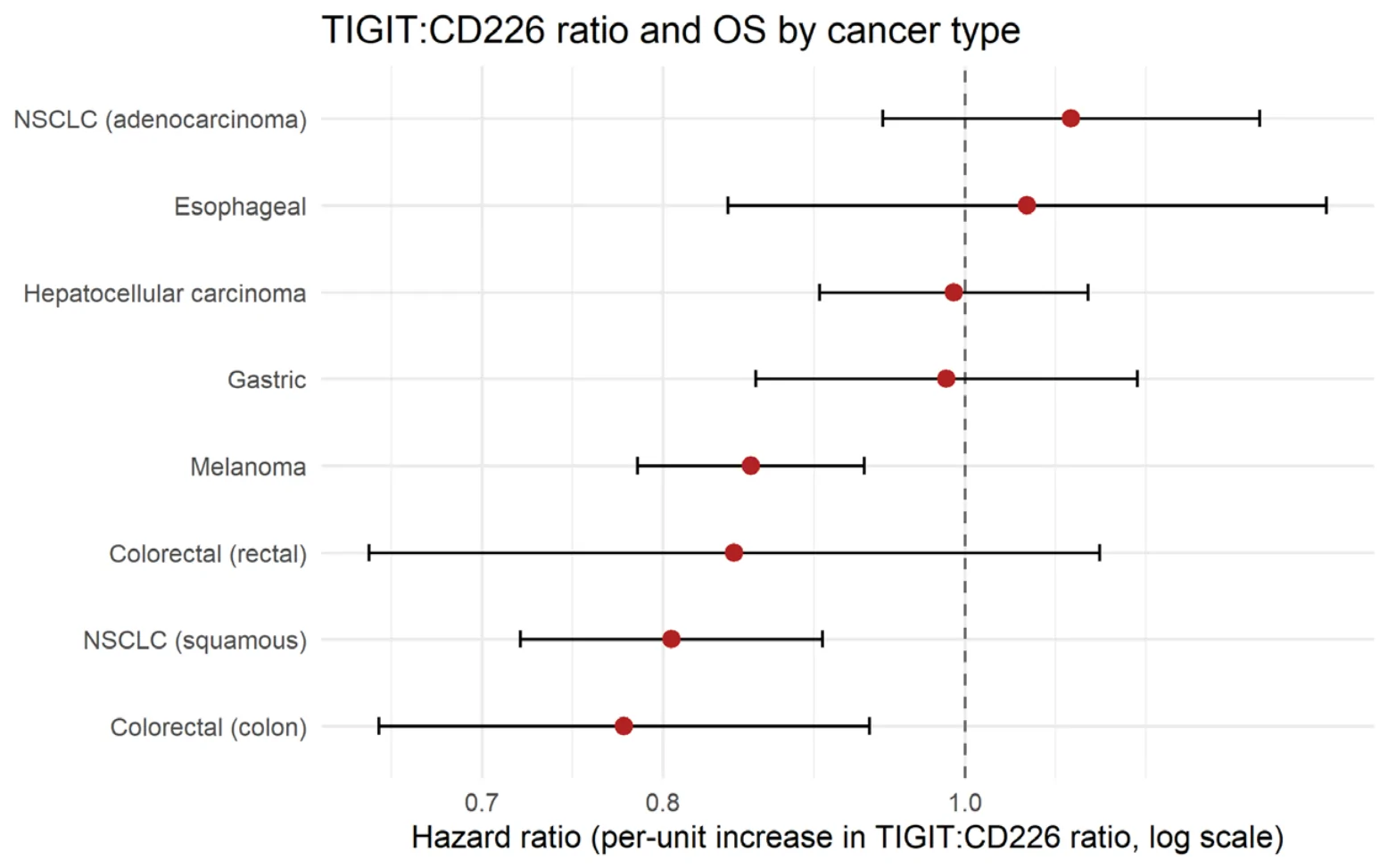
Forest plot of hazard ratios (univariate Cox regression) for overall survival per unit increase in the log_2_ *TIGIT*:*CD226* expression ratio, across eight TCGA cancer types (*n* = 3235 samples with complete survival data). HR < 1 (left of the dashed reference line) indicates that a higher *TIGIT*:*CD226* ratio is associated with improved survival. *TIGIT*, t-cell immunoreceptor with Ig and ITIM domains; *CD226*, cluster of differentiation 226; OS, overall survival; NSCLC, non-small cell lung cancer; HR, hazard ratio; and CI, confidence interval.

**Figure 6:**
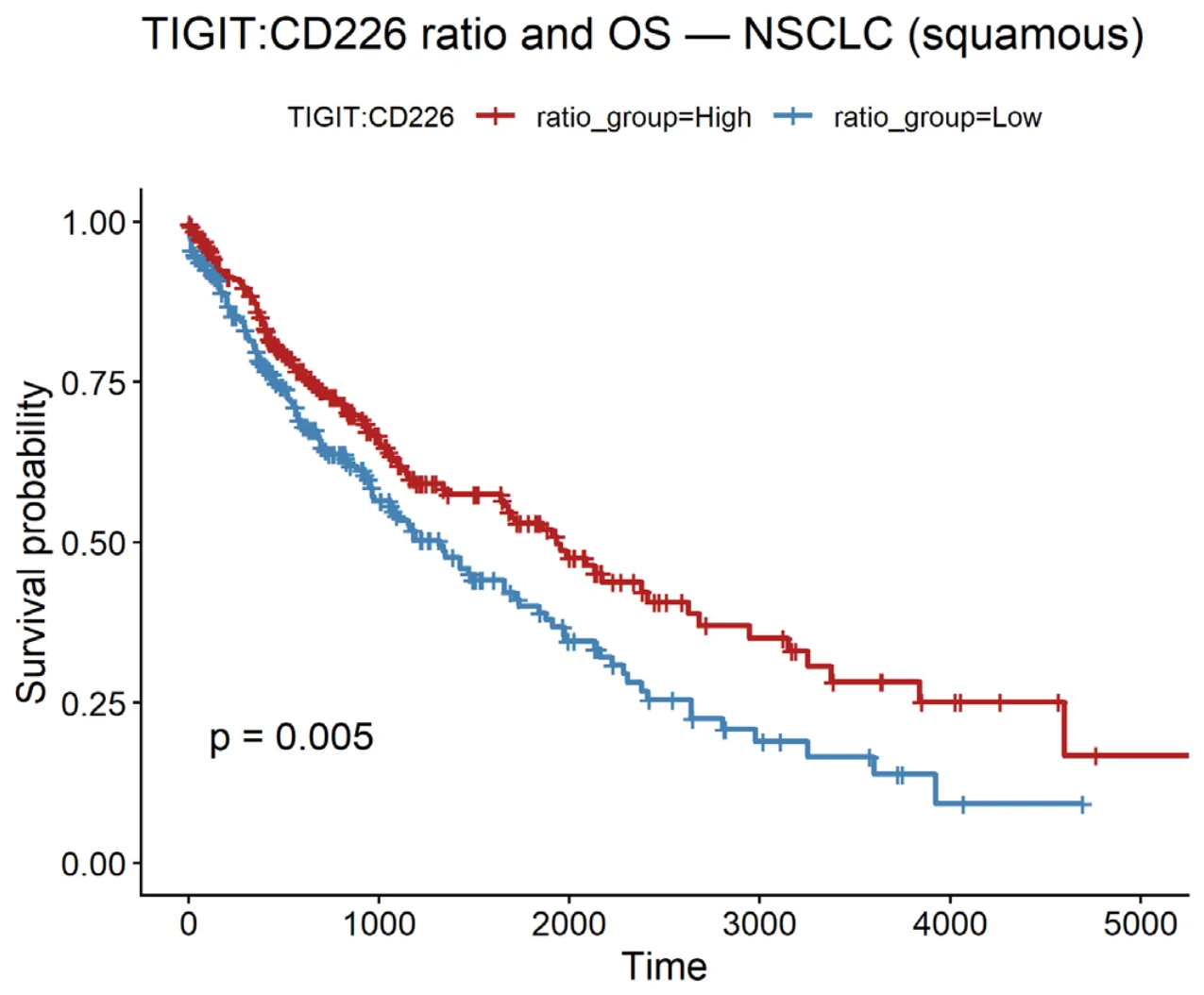
Kaplan–Meier overall survival curves stratified by median-dichotomized TIGIT:CD226 ratio in lung squamous cell carcinoma (TCGA-LUSC, *n* = 544), the cancer type with the strongest survival association in the pan-cancer analysis (log-rank *p* = 0.005). *TIGIT*, t-cell immunoreceptor with Ig and ITIM domains; *CD226*, cluster of differentiation 226 (DNAX accessory molecule-1, DNAM-1); OS, overall survival; NSCLC, non-small cell lung cancer; and *p*, probability value.

Across all eight cancer types examined in the TCGA Pan-Cancer Atlas, the median log_2_ *TIGIT*:*CD226* expression ratio was positive (Figure 3), indicating that *TIGIT* transcript abundance exceeds that of its competing activating receptor *CD226* essentially universally in the tumor immune microenvironment. The gene-level heatmap (Figure 4) further shows that this pattern is not uniform across the checkpoint panel: NSCLC (both adenocarcinoma and squamous subtypes) displayed broad elevation across nearly all seven genes examined, whereas hepatocellular carcinoma and melanoma showed more selective, gene-specific patterns, for example, hepatocellular carcinoma combined low *TIGIT*, *PDCD1*, and *LAG3* expression with near-neutral *CD226* and *CD28* expression. This heterogeneity indicates that the checkpoint co-expression architecture, not merely the *TIGIT*:*CD226* ratio, differs meaningfully by tumor lineage. In univariate Cox regression, a higher *TIGIT*:*CD226* ratio was associated with significantly improved OS in colon adenocarcinoma (hazard ratio (HR) = 0.78, 95% confidence interval (CI) 0.65–0.93, *p* = 0.006), melanoma (HR = 0.85, 95% CI 0.79–0.93, *p* < 0.001), and NSCLC-squamous carcinoma (HR = 0.80, 95% CI 0.72–0.90, *p* < 0.001), but not in the remaining five cancer types (Figure 5 and Figure 6). This unadjusted, univariate association, opposite in direction to a simple “more checkpoint expression is worse” interpretation, illustrates that the prognostic meaning of the *TIGIT*:*CD226* axis is context-dependent and cannot be assumed to generalize across tumor types.

### 4.2. Validation in Immune-Checkpoint-Blockade-Treated Cohorts

To test whether the TIGIT:CD226 axis has predictive value for treatment outcome, rather than purely descriptive value in treatment-naive tumors, we analyzed two independent, publicly available cohorts of patients treated with anti-PD-1/PD-L1 monotherapy with pre-treatment RNA-sequencing and documented clinical response: (i) GSE91061, 49 patients with metastatic melanoma treated with nivolumab with baseline tumor biopsies [56]; and (ii) the IMvigor210 cohort, 298 patients with locally advanced or metastatic urothelial carcinoma treated with atezolizumab with baseline tumor RNA-sequencing and annotated best confirmed overall response and overall survival [57]. These two tumor types were selected because they represent the only public, individual-patient-level, response-annotated RNA-sequencing datasets currently available for anti-PD-L1 monotherapy; equivalent response-linked transcriptomic data are not yet publicly available for NSCLC or gastrointestinal malignancies. The same seven-gene panel and composite score described above were computed for each cohort. Patients were classified as responders (complete or partial response, RECIST) or non-responders (stable or progressive disease); patients with indeterminate response were excluded. Group differences were assessed by two-sided Wilcoxon rank-sum test; survival association was assessed by log-rank test. For the 42 GSE91061 patients with both pre-treatment and on-treatment biopsies, we additionally computed the on-treatment change (Δ) in TIGIT:CD226 ratio and tested its association with response, as a secondary, pharmacodynamic analysis distinct from the baseline-predictive analyses above.

**Figure 7:**
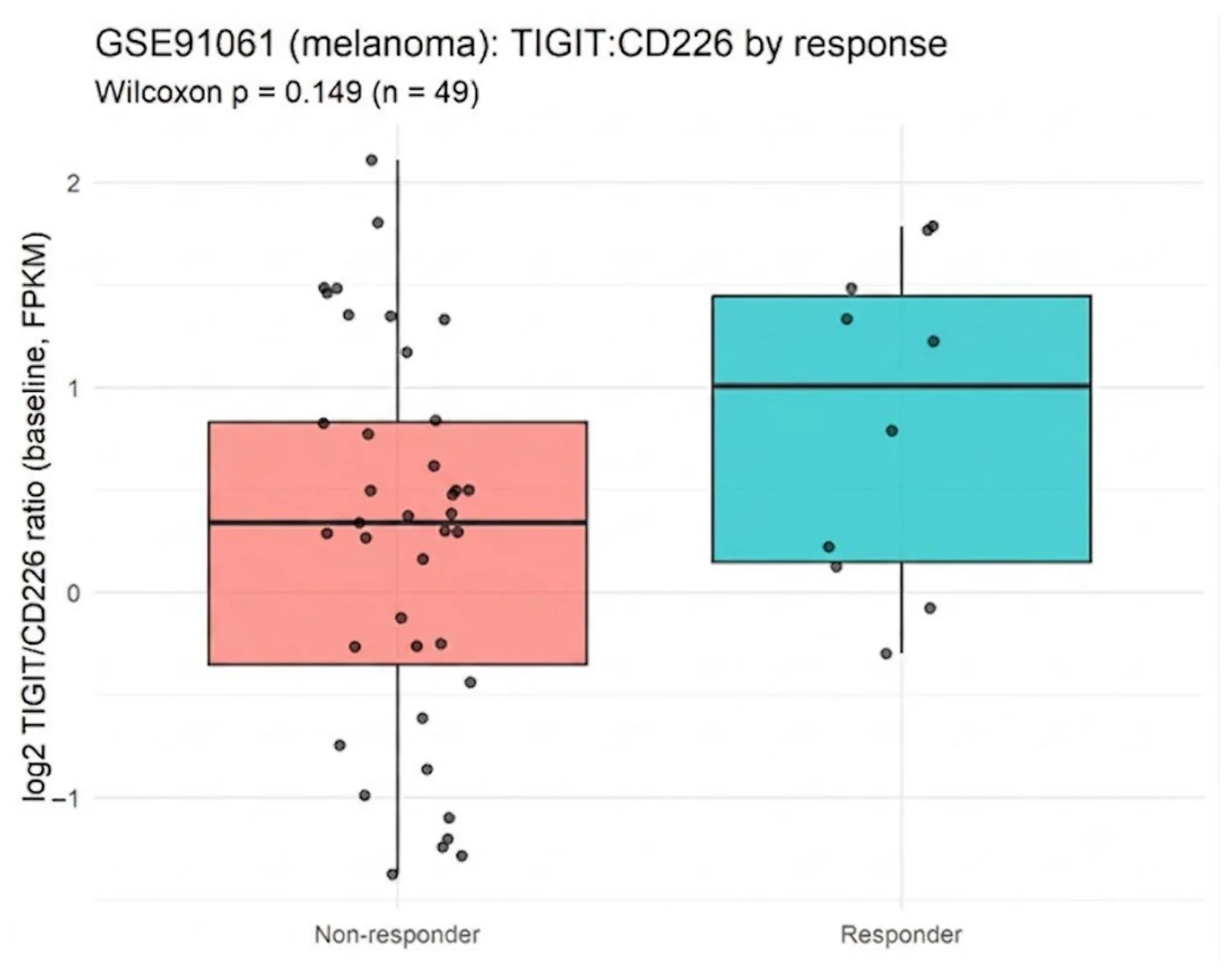
Baseline (pre-treatment) log_2_ *TIGIT*:*CD226* expression ratio by objective response (RECIST) to nivolumab in patients with metastatic melanoma (GSE91061, *n* = 49). Wilcoxon rank-sum *p* = 0.149. *TIGIT*, t-cell immunoreceptor with Ig and ITIM domains; *CD226*, cluster of differentiation 226; FPKM, fragments per kilobase of transcript per million mapped reads; and *p*, probability value.

**Figure 8:**
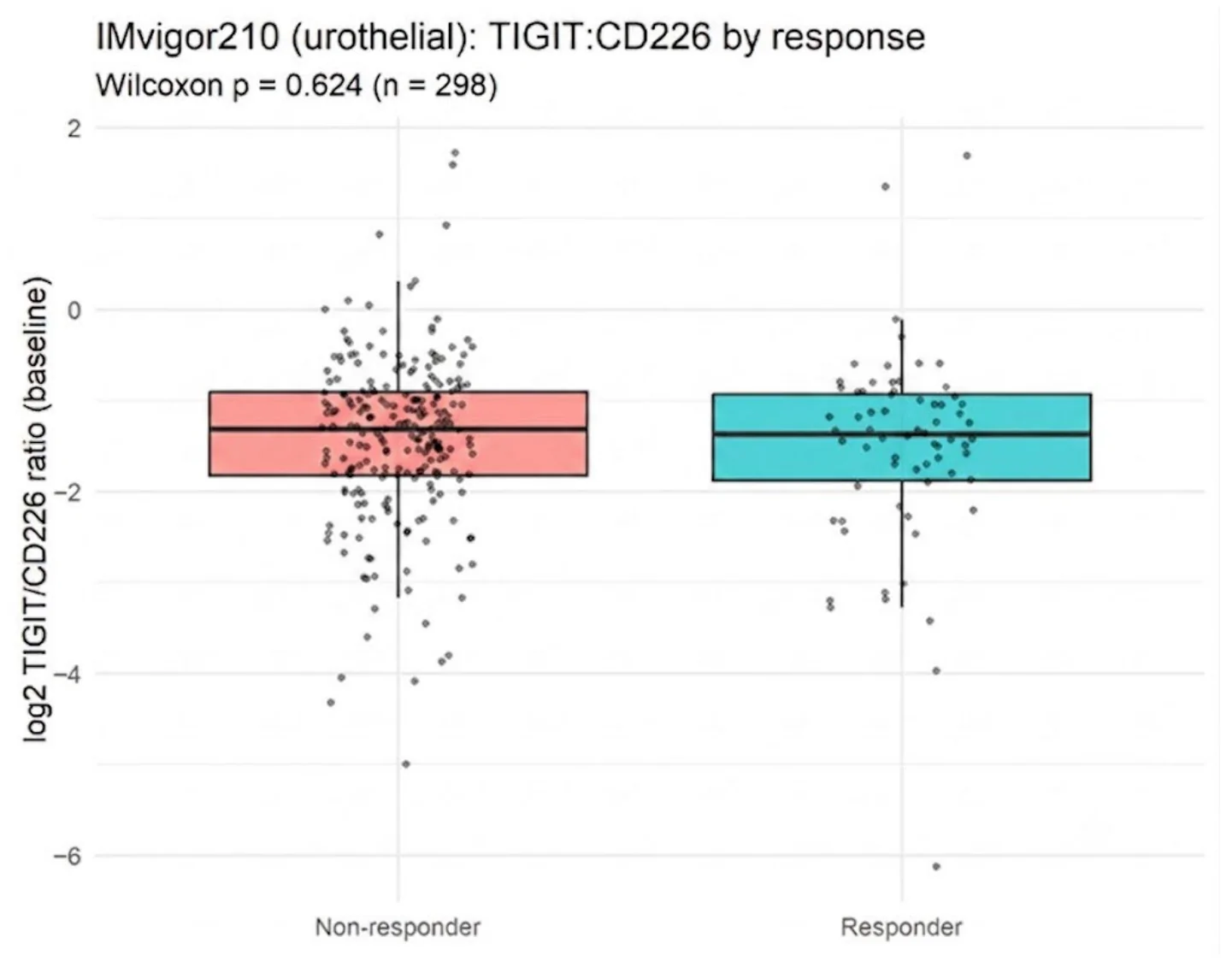
Baseline log_2_ TIGIT:CD226 expression ratio by objective response to atezolizumab in patients with locally advanced or metastatic urothelial carcinoma (IMvigor210, *n* = 298). Wilcoxon rank-sum *p* = 0.624. *TIGIT*, t-cell immunoreceptor with Ig and ITIM domains; *CD226*, cluster of differentiation 226; and *p*, probability value.

**Figure 9:**
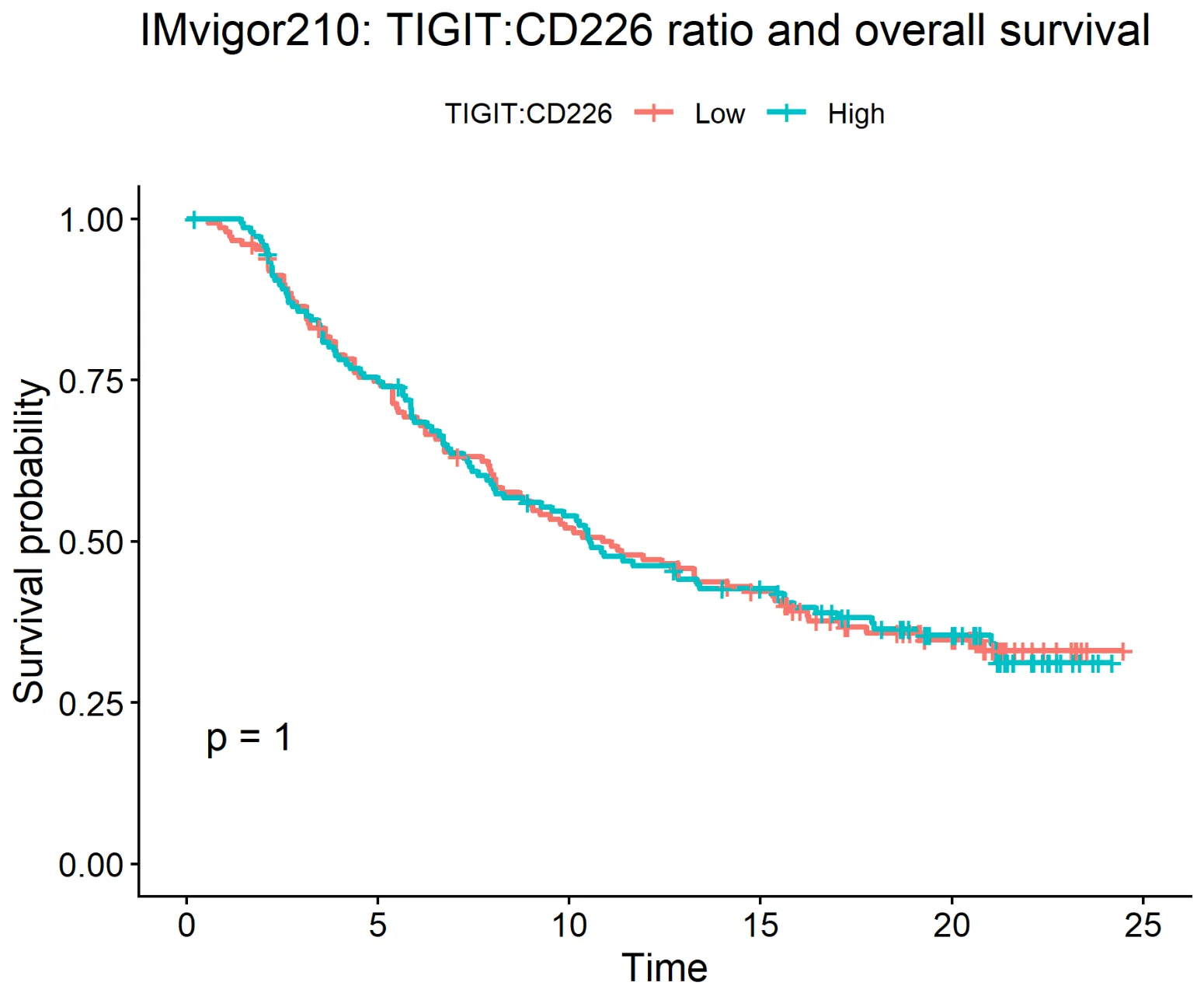
Kaplan–Meier overall survival curves stratified by median-dichotomized baseline *TIGIT*:*CD226* ratio in the IMvigor210 cohort (*n* = 298). Log-rank *p* = 1.0. *TIGIT*, t-cell immunoreceptor with Ig and ITIM domains; *CD226*, cluster of differentiation 226 (DNAX accessory molecule-1, DNAM-1); OS, overall survival; and *p*, probability value.

**Figure 10:**
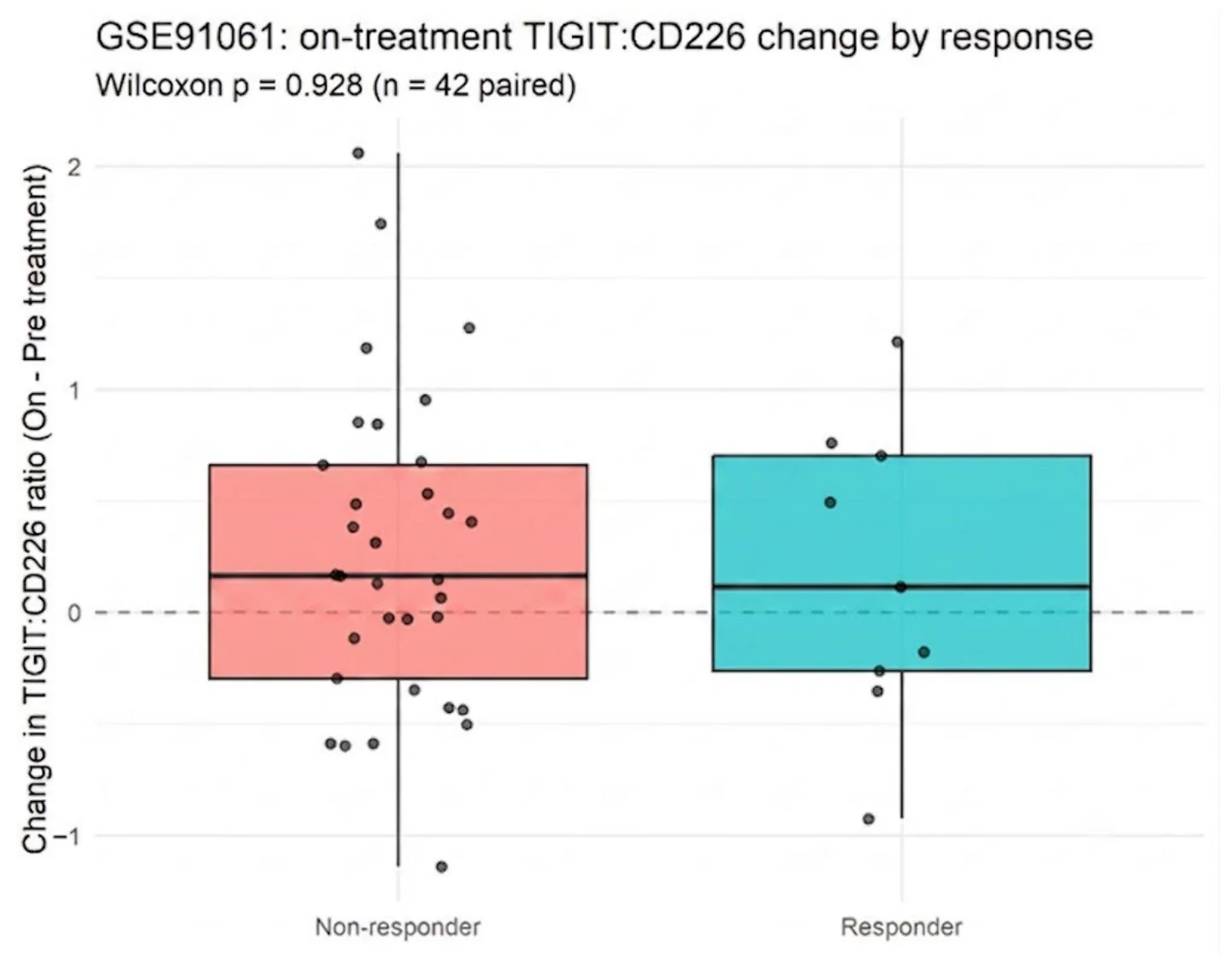
On-treatment change (Δ, on-treatment minus pre-treatment) in log_2_ TIGIT:CD226 ratio by objective response, in the subset of GSE91061 patients with paired pre- and on-treatment biopsies (*n* = 42). Wilcoxon rank-sum *p* = 0.928. *TIGIT*, t-cell immunoreceptor with Ig and ITIM domains; *CD226*, cluster of differentiation 226; and *p*, probability value.

All analyses were performed in R (v4.6.1). Statistical significance was set at *p* < 0.05, two-sided, without correction for multiple comparisons across the eight cancer types tested in the pan-cancer survival screen; this exploratory, hypothesis-generating framing is retained throughout the Results below. In contrast to its cancer-type-dependent prognostic associations in treatment-naive TCGA tumors, the baseline *TIGIT*:*CD226* ratio was not significantly associated with objective response to checkpoint blockade in either the melanoma/nivolumab cohort (GSE91061, *n* = 49; Wilcoxon *p* = 0.149; Figure 7) or the substantially larger and better-powered urothelial/atezolizumab cohort (IMvigor210, *n* = 298; Wilcoxon *p* = 0.624; Figure 8), nor with OS in the latter (log-rank *p* = 1.0; Figure 9). In the subset of 42 GSE91061 patients with paired pre- and on-treatment biopsies, the on-treatment change in *TIGIT*:*CD226* ratio likewise showed no association with response (Wilcoxon *p* = 0.928; Figure 10), indicating that neither the baseline level nor the early pharmacodynamic change in this two-gene ratio is sufficient, on its own, to stratify likely responders.

### 4.3. Composite Checkpoint-Balance Score Improves Response Association

**Figure 11:**
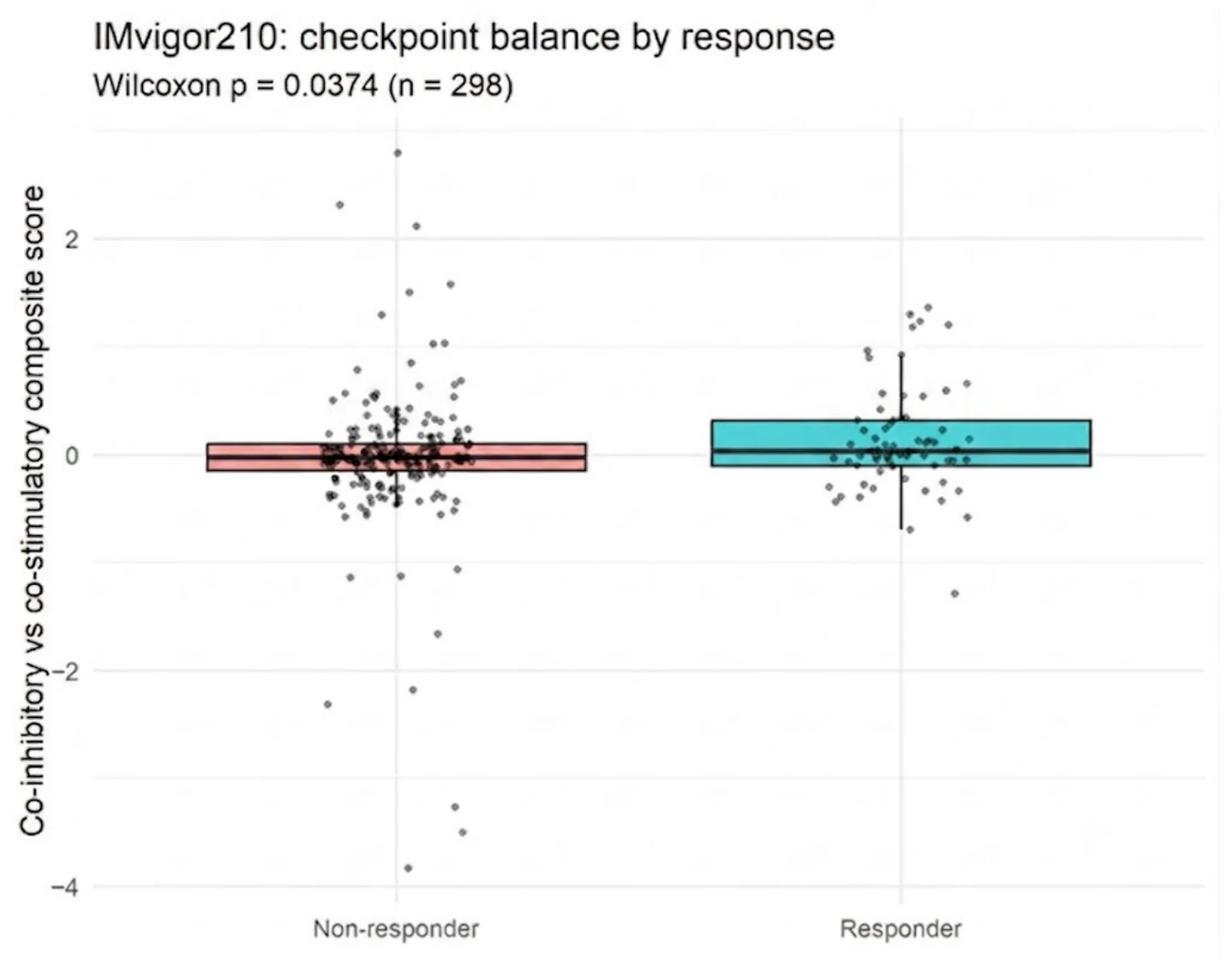
Baseline composite co-inhibitory (*TIGIT*, *PDCD1*, *LAG3*, and *HAVCR2*) versus co-stimulatory (*CD226*, *CD28*, and *ICOS*) checkpoint balance score by objective response to atezolizumab in the IMvigor210 cohort (*n* = 298). Wilcoxon rank-sum *p* = 0.037. *TIGIT*, t-cell immunoreceptor with Ig and ITIM domains; *PDCD1*, programmed cell death 1; *LAG3*, lymphocyte activation gene 3; *HAVCR2*, hepatitis A virus cellular receptor 2; *CD226*, cluster of differentiation 226; *CD28*, cluster of differentiation 28; *ICOS*, inducible T-cell costimulator; and *p*, probability value.

When the analysis was extended from the isolated TIGIT:CD226 ratio to the seven-gene composite score incorporating PDCD1, LAG3, and HAVCR2 alongside TIGIT (co-inhibitory axis) and CD28 and ICOS alongside CD226 (co-stimulatory axis), responders in the IMvigor210 cohort showed significantly higher baseline co-inhibitory dominance than non-responders (median composite score 0.03 versus −0.03; Wilcoxon *p* = 0.037; Figure 11), although this did not translate into a significant OS difference (log-rank *p* = 0.2). The same composite score was not significant in the smaller GSE91061 cohort (Wilcoxon *p* = 0.371), consistent with its more limited statistical power (*n* = 49 versus *n* = 298).

### 4.4. Gene-Level Contributions to the Composite Score

**Figure 12:**
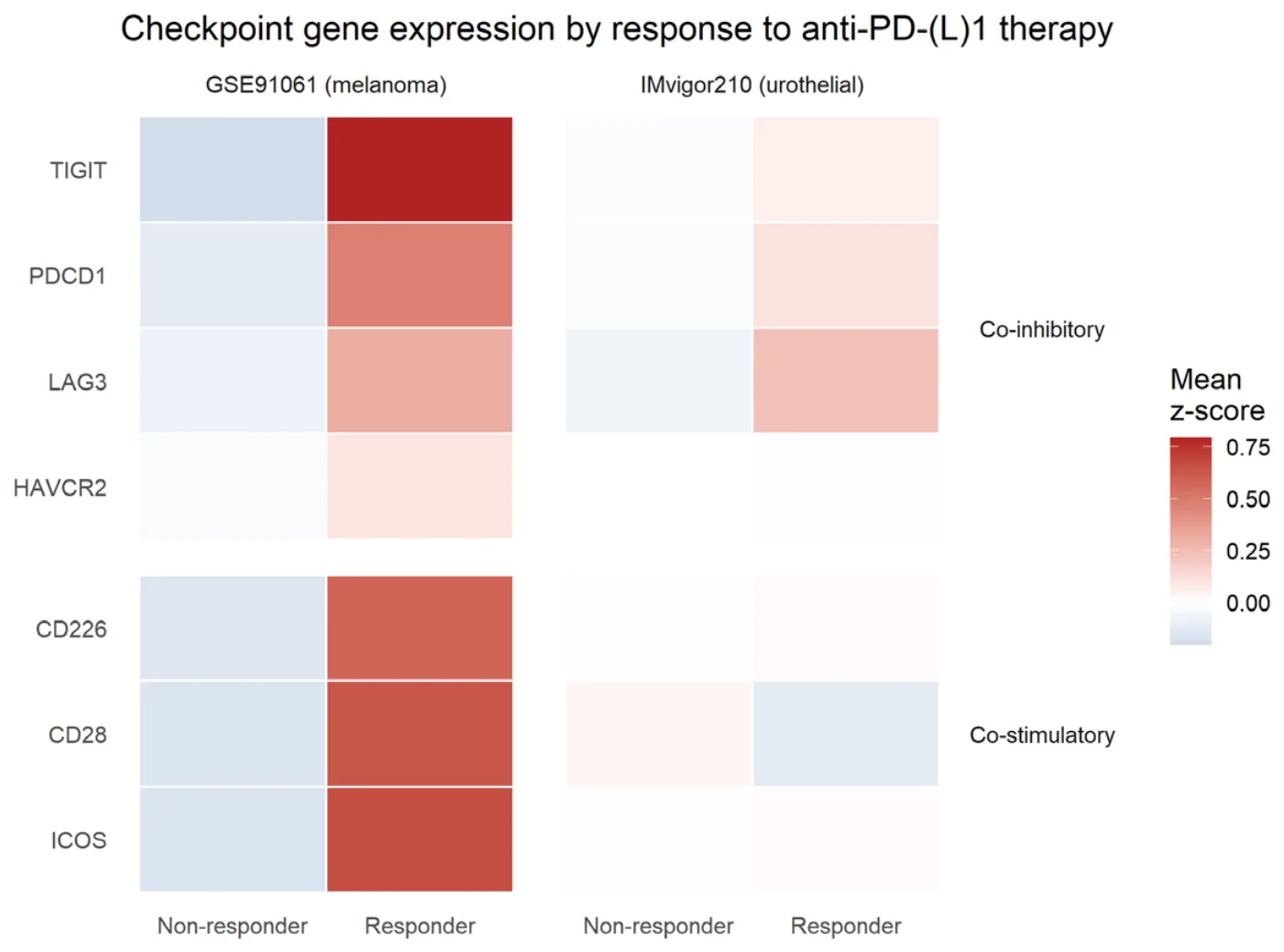
Heatmap of mean z-score expression for the same seven checkpoint genes, stratified by objective response, in the GSE91061 (melanoma/nivolumab, *n* = 49) and IMvigor210 (urothelial/atezolizumab, *n* = 298) cohorts. *TIGIT*, t-cell immunoreceptor with Ig and ITIM domains; *PDCD1*, programmed cell death 1; *LAG3*, lymphocyte activation gene 3; *HAVCR2*, hepatitis A virus cellular receptor 2; *CD226*, cluster of differentiation 226; *CD28*, cluster of differentiation 28; *ICOS*, inducible T-cell costimulator; PD-(L)1, programmed cell death protein-1 (ligand-1); and z-score, standard score.

Gene-level heatmaps of the responder and non-responder groups in each cohort (Figure 12) offer a mechanistic explanation for this pattern. In GSE91061, responders showed broadly elevated expression across essentially all seven checkpoint genes examined, including both co-inhibitory and co-stimulatory members; because a ratio or balance score is constructed by subtraction between these two groups, a uniform, non-differential elevation across both axes is not well captured by either metric, which likely explains why neither the *TIGIT*:*CD226* ratio nor the composite score reached significance in this cohort despite visibly higher absolute expression in responders. In IMvigor210, by contrast, the gene-level pattern was more differential: *LAG3* showed the strongest and most consistent separation between responders and non-responders of the entire seven-gene panel, with more modest elevation of *TIGIT* and *PDCD1* and negligible difference in *CD226*, *CD28*, or *ICOS*. This indicates that *LAG3*, rather than *TIGIT* itself, was the principal contributor to the significant composite-score association reported above, and suggests that the co-inhibitory receptor most informative for patient stratification may vary by tumor type and treatment context rather than being fixed to *TIGIT* across settings.

These original analyses demonstrate empirically that (i) the *TIGIT*:*CD226* relationship and the broader checkpoint co-expression pattern carry cancer-type-specific prognostic information in treatment-naive tumors; (ii) a two-gene ratio alone is insufficient to predict response to checkpoint blockade in two independent, real-world treated cohorts; and (iii) a broader multi-receptor composite score captures response-associated signal that the isolated ratio misses, with the identity of the most informative individual gene (*LAG3* in IMvigor210) itself varying by context. This pattern provides direct, data-driven support for the central argument of this review, that meaningful biomarker development for TIGIT-directed and combination immunotherapy requires integration across the co-inhibitory and co-stimulatory checkpoint network, rather than reliance on *TIGIT* expression in isolation.

## 5. Immunotherapy Agents Targeting TIGIT: Preclinical and Clinical Evidence

### 5.1. Pipeline of Anti-TIGIT Monoclonal Antibodies in Clinical Development

**Figure 13:**
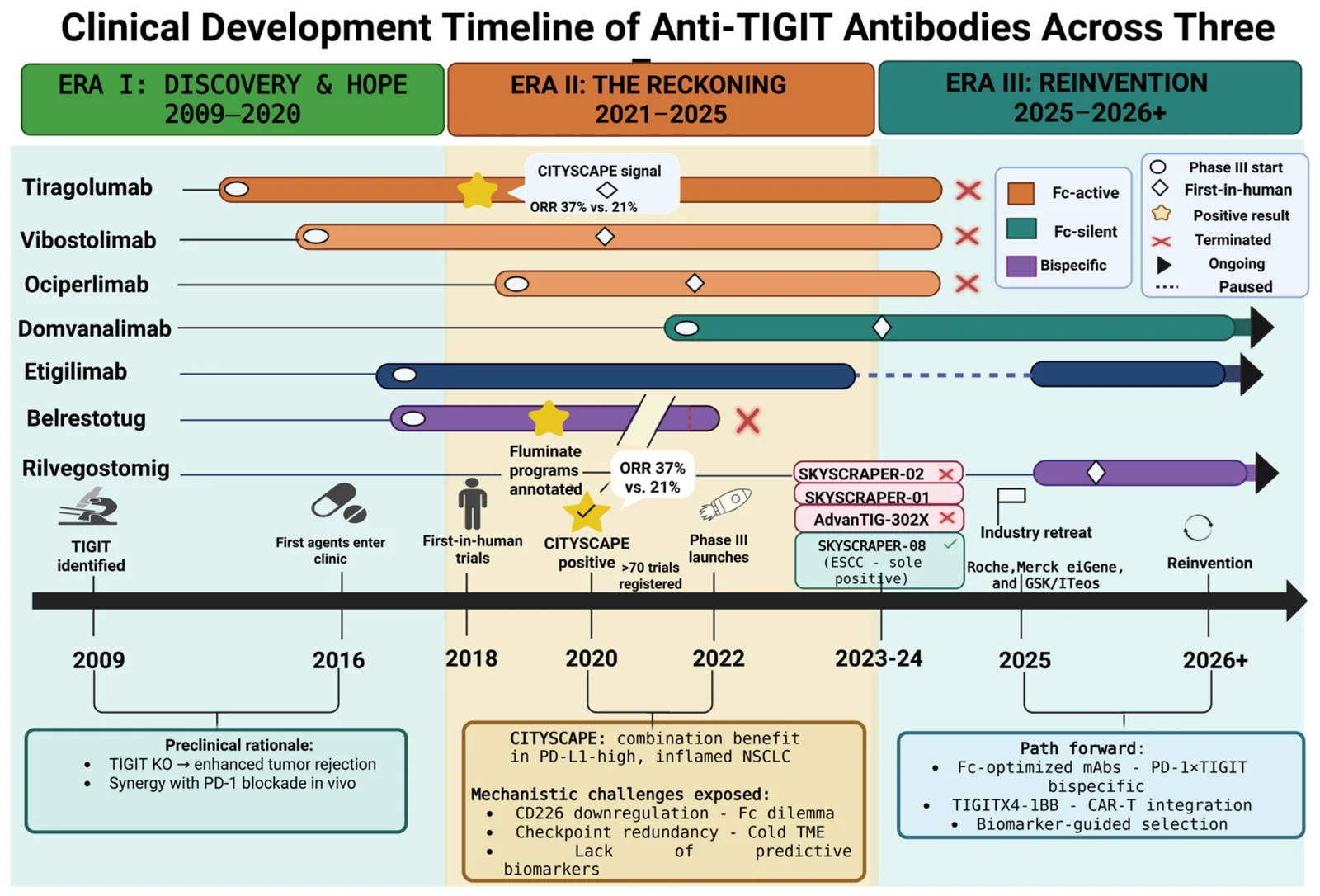
Clinical Development Timeline of Anti-TIGIT Antibodies Across Three Eras (2009–2026+). Comprehensive longitudinal swim-lane timeline tracking 7 drugs across three eras. ERA I (2009–2020, Discovery & Hope): TIGIT identified, first agents in clinic, CITYSCAPE positive (ORR 37% vs. 21% in PD-L1-high subgroup; 31.3% vs. 16.2% overall), >70 trials registered. ERA II (2021–2025, The Reckoning): Tiragolumab, Vibostolimab, Ociperlimab, and Belrestotug all terminated following Phase III failures (SKYSCRAPER-01/02, AdvanTIG-302); mechanistic barriers exposed (CD226 downregulation, Fc dilemma, checkpoint redundancy, no biomarkers); industry retreat by Roche, Merck, BeiGene, GSK/iTeos. ERA III (2025–2026+, Reinvention): Domvanalimab (Fc-silent, ongoing) and Rilvegostomig (PD-1 × TIGIT bispecific, ongoing) lead the next generation, supported by Fc-optimized mAbs, TIGITx4-1BB designs, CAR-T integration, and biomarker-guided selection. TIGIT; PD-1, programmed cell death protein 1; PD-L1, programmed death-ligand 1; ORR, overall response rate; NSCLC, non-small cell lung cancer; Fc, fragment crystallizable region; CAR-T, chimeric antigen receptor T-cell; TME, tumor microenvironment; ESCC, esophageal squamous cell carcinoma. Thin horizontal lines leading to each bar represent preclinical development prior to clinical initiation. The red vertical dashed line indicates [insert exact meaning, e.g., temporary trial halt/program split]. Green checkmarks denote positive trial outcomes meeting primary endpoints. Created in BioRender. Smail, S. (2026) https://BioRender.com/b4fanvf.

The clinical pipeline comprises both monospecific anti-TIGIT antibodies and TIGIT-containing bispecific antibodies (Table 3, Figure 13), including tiragolumab [40,58,59,60], vibostolimab [61,62,63,64,65], ociperlimab [24,66], domvanalimab [67,68], etigilimab [20,69,70], EOS-448 (GSK4428859A; belrestotug) [71,72,73,74], COM902 [75], M6223 [76,77] and the PD-1 × TIGIT bispecific rilvegostomig [78,79,80,81]. These agents represent distinct antibody-engineering strategies, ranging from Fc-competent or Fc-active IgG1 molecules to Fc-enhanced and Fc-silent formats, as well as IgG4 antibodies designed to retain minimal effector function. Their intended mechanisms consequently differ: immune-checkpoint blockade may be combined with FcγR-mediated immune modulation, ADCC, selective depletion of TIGIT-positive regulatory T cells, antigen-presenting-cell or myeloid activation, and reprogramming of exhausted CD8^+^ T cells toward a more functional state [40,66,67,73,74,75,76,77]. The clinical experience has been heterogeneous. Tiragolumab, vibostolimab and ociperlimab have produced disappointing Phase III outcomes or undergone program discontinuation [23,24,62,63]. By contrast, domvanalimab, etigilimab, EOS-448/GSK4428859A, COM902, M6223 and rilvegostomig remain under clinical investigation. Across the pipeline, efficacy and safety vary with Fc configuration and the biological context in which TIGIT is targeted, including the combination partner—particularly PD-1 or PD-L1 blockade—the tumor type and patient-selection factors such as PD-L1 expression [13,19,45,46]. Table 3 summarizes the corresponding clinical findings, safety profiles and development status without implying that any single antibody format or mechanism is uniformly superior.

### 5.2. Preclinical Studies and Mechanistic Insights

Preclinical mouse Tumor models have provided foundational findings demonstrating that TIGIT functions as a crucial inhibitory checkpoint that limits anti-tumor immunity [82]. Genetic deletion research using Tigit^−/−^ mice demonstrated enhanced immune-mediated tumor suppression across multiple syngeneic Tumor models, including melanoma (B16), colon cancer (MC38), and fibrosarcoma. The lack of TIGIT in these mice resulted in increased infiltration and functional recovery of CD8^+^ T cells and NK cells, marked by elevated IFN-γ production, augmented granzyme B expression, and greater cytotoxicity against tumor cells. The lack of TIGIT did not lead to noticeable systemic autoimmunity, suggesting a therapeutically promising avenue for TIGIT targeting (Table 4) [14,16,17].

Investigations on antibody-mediated TIGIT suppression corroborated the genetic findings and revealed important mechanistic insights [83]. In animal tumor models, administration of anti-TIGIT monoclonal antibodies alone led to a modest postponement in tumor proliferation. The concurrent inhibition of TIGIT and PD-1 or PD-L1 frequently exhibited synergistic anti-tumor effects, including prolonged tumor regression and improved survival [18]. The results were mechanistically linked to the reestablishment of CD226 co-stimulatory signaling on CD8^+^ T cells and NK cells, which is otherwise suppressed by TIGIT engagement. The data validated TIGIT as a distinct checkpoint that collaborates with PD-1 to induce T-cell exhaustion, providing a rationale for the integration of immunotherapeutic approaches in the laboratory [14].

Preclinical investigations have revealed a pivotal role of TIGIT in sustaining Treg cell-mediated immunosuppression, in addition to its activity in effector cells [84]. Research involving murine models indicated that TIGIT^+^ Tregs exhibit superior suppressive capabilities compared to TIGIT^−^ Tregs, particularly in the context of malignancies [40]. TIGIT signaling enhanced the stability of Tregs, maintained elevated *forkhead box P3* (*FOXP3*) expression, and augmented IL-10 secretion, hence facilitating immunological tolerance within the TME. Significantly, the inhibition of TIGIT solely destabilized intratumoral Tregs, leaving peripheral Tregs unaffected, therefore altering the equilibrium between effector and regulatory cells to promote tumor rejection [85].

Mechanistic analysis in mouse models clarified the importance of TIGIT ligand interactions in regulating the tumor immune microenvironment. Tumor cells and tumor-associated myeloid cells frequently exhibit elevated levels of CD155, which preferentially binds to TIGIT over the activating receptor CD226 [4,86]. In vivo studies demonstrated that disrupting the TIGIT–CD155 axis not only restored the functionality of cytotoxic lymphocytes but also transformed DCs into a more immunostimulatory phenotype, evidenced by elevated levels of IL-12 and reduced levels of IL-10. This dual mechanism, both intrinsic and extrinsic to the cell, demonstrates how TIGIT inhibition amplifies anti-tumor immunity at several levels inside the TME [19,87].

**Table 4:** TIGIT inhibitors evaluated in preclinical studies, organized by modality.

Agent/Approach	Type	Developer/Group	Tumor Models	Key Preclinical Findings
Anti-mouse TIGIT mAb (313R12)	Surrogate mouse mAb (Fc-active, IgG2a)	Academic/research antibody; murine TIGIT studies	CT26 colon, Renca kidney	Single-agent TGI in multiple syngeneic models; complete tumor rejection + durable immunologic memory when combined with anti-PD-1 or anti-PD-L1; enhanced Th1 responses and CTL function [88].
313R12 (biomarker/MOA study)	Same as above	Academic preclinical research tool antibody	CT26 colon	Treg loss from tumors within 24 h; sustained through 14 days. CD226 upregulation on T/NK cells. Effector-function competent (IgG2a) was required for TGI—Fc-deficient variant (313R13) lost efficacy [89].
Ociperlimab (BGB-A1217)	Humanized IgG1 mAb (Fc-competent)	BeiGene (BeOne Medicines)	Syngeneic models in human TIGIT-KI mice	High-affinity binding (KD = 0.135 nM); blocks TIGIT–CD155/CD112; induces ADCC against Tregs, activates NK cells. In vivo: potent antitumor efficacy alone and with anti-PD-1; Fc effector function critical for activity [90].
T4 antibody	Cross-species (human/mouse) mAb	Academic preclinical anti-TIGIT antibody	B16, MC38, CT26, EMT6	Binds human and mouse TIGIT; strong antitumor activity + durable cross-tumor immune memory. Fc-dependent Treg depletion via NK cells; Fc-enhanced variants showed further improved efficacy [91].
SEA-TGT surrogate (Seagen)	Effector-function enhanced mAb	Seagen (Pfizer)	CT26, MC38, A20	Treg depletion via ADCC; up to 66% complete responses in A20 model; curative responses with long-term memory; activated both innate and adaptive immunity [92].
EOS884448 surrogate	Fully human IgG1 mAb (ADCC-active)	iTeos Therapeutics	Mouse syngeneic models	Potent antitumor activity via antagonism + Fc’γR engagement; Treg depletion via ADCC; safe toxicology in cynomolgus monkeys (≤10 mg/kg) [93].
AB154 (domvanalimab)/AB308 surrogates	Fc-silent (AB154) vs. Fc-active (AB308) mAbs	Arcus Biosciences; Arcus/AstraZeneca collaboration	Syngeneic mouse models	Both enhanced tumor control when combined with anti-PD-1; Fc-active variant associated with intratumoral Treg depletion. Targeted tumor-reactive and stem-like CD8^+^ T cells [94].
Tiragolumab in HuGEMM models	Humanized IgG1 mAb (Roche)	Genentech/Roche	Hepa 1–6, CT26 in TIGIT-KI mice	>90% TGI in Hepa 1–6 monotherapy; 41% TGI monotherapy → 68% TGI with anti-PD-1 combo in CT26; increased CD8^+^ T cell infiltration with combination [95].
TIGIT blockade + Flt3L	Anti-TIGIT mAb + Flt3L gene delivery	Academic/preclinical approach	B16/F10 melanoma, colon, breast, fibrosarcoma	Landmark study: TIGIT blockade reversed NK cell exhaustion; combined with Flt3L, suppressed established tumor growth and metastasis; improved survival across multiple models. TIGIT identified as key NK cell checkpoint [96].
αTIGIT + bintrafusp alfa	Anti-TIGIT + PD-L1/TGFβ trap (triple pathway)	EMD Serono/Merck KGaA and GSK	MC38-CEA (ICB-resistant), TC-1	Significant antitumor activity even in ICB-resistant models; complete responses with durable memory; dependent on CD4^+^ and CD8^+^ T cells [97].
TIGIT + PD-1 blockade + RT	Anti-TIGIT mAb + anti-PD-1 + radiation	Academic/preclinical combination strategy	Murine TNBC	Triple combination synergistic: increased CD8^+^ TIL infiltration in irradiated and non-irradiated tumors, reduced Tregs, less exhausted T cell phenotype [98].
HB0036	PD-L1 × TIGIT bispecific Ab	Biotheus Inc.	Syngeneic + xenograft models	Greater T-cell proliferation than combo of parental Abs; enriched TIGIT Ab at PD-L1+ tumors; improved tumor control. Adding anti-VEGF further enhanced efficacy [99].
TIGIT/PD-L1 co-blockade (Genentech MOA)	Anti-TIGIT + anti-PD-L1	Genentech/Roche	CT26	Required lymphocyte trafficking between dLN and tumor; promoted clonal expansion of non-exhausted CD8^+^ T cells; decreased Tox expression; expanded memory-like T cells [100].
TIGIT KO NK cells	CRISPR-Cas9 KO in expanded NK cells	Academic/research antibody; murine TIGIT studies	A549, NCI-H1299 (lung cancer spheroids)	TIGIT KO increased NK cytotoxicity, upregulated mTORC1 signaling, improved metabolic fitness. Critically, prevented NK fratricide when combined with Fc-active anti-TIGIT Abs [101].
D-peptide DTBP-3	D-enantiomer peptide (mirror-image phage display)	Academic preclinical research tool antibody	MC38, anti-PD-1 resistant models	First D-peptide targeting TIGIT; blocks TIGIT–CD155; proteolytic resistance + tumor penetration; suppressed tumors in CD8^+^ T cell–dependent manner; active in anti-PD-1 resistant model [102].
Gln(TrT)	Dual TIGIT/PD-1 small molecule	BeiGene (BeOne Medicines)	MC38	First reported small molecule blocking both TIGIT/CD155 and PD-1/PD-L1; restored Jurkat T-cell function in vitro; promoted intratumoral CD8^+^ T cell infiltration in vivo [103].
INTASYL RNAi (PH-804)	Self-delivering RNAi (intratumoral)	Academic preclinical anti-TIGIT antibody	CT26 colon	~90% TIGIT mRNA knockdown; combination with PD-1/PD-L1 INTASYL improved tumor control vs. monotherapy; intratumoral delivery may limit systemic irAEs [104].
Natural compound screen	In silico small molecule candidates (bacterial origin)	Seagen (Pfizer)	Computational only	Virtual screening identified 6 bacterial-derived candidates (e.g., Neomycin K, Zwittermicin A) predicted to block TIGIT–CD155; awaiting experimental validation [105].

mAb, monoclonal antibody; Fc, fragment crystallizable region; FcγR, Fc gamma receptor; IgG, immunoglobulin G; IgG1/IgG2a, immunoglobulin G subclasses 1 and 2a; KD, equilibrium dissociation constant; KI, knock-in; TGI, tumor growth inhibition; CTL, cytotoxic T lymphocyte; Th1, T-helper type 1; Treg, regulatory T cell; NK cells, natural killer cells; ADCC, antibody-dependent cellular cytotoxicity; PD-1, programmed cell death protein 1; PD-L1, programmed death-ligand 1; TILs, tumor-infiltrating lymphocytes; dLN, draining lymph node; TNBC, triple-negative breast cancer; RT, radiotherapy; VEGF, vascular endothelial growth factor; KO, knockout; CRISPR, clustered regularly interspaced short palindromic repeats; RNAi, RNA interference; irAEs, immune-related adverse events; MOA, mechanism of action; HuGEMM, humanized genetically engineered mouse model.

### 5.3. Clinical Trials Targeting TIGIT

The clinical application of TIGIT blockade has progressed rapidly in the past decade, highlighting its role as a distinct immune checkpoint and its ability to enhance antitumor immunity, particularly when used alongside PD-1/PD-L1 inhibitors (Table 5) [20]. The preliminary stage of clinical development focused on assessing the safety, pharmacokinetics, and preliminary anticancer activity of anti-TIGIT monoclonal antibodies in advanced solid tumors. The GO30103 Phase 1a/1b trial evaluated the anti-TIGIT antibody tiragolumab as both a monotherapy and in combination with the PD-L1 inhibitor atezolizumab in patients with advanced solid tumors. This trial demonstrated that tiragolumab was well tolerated and that its combination with atezolizumab exhibited preliminary indications of antitumor efficacy, with objective responses observed in PD-1/PD-L1–naïve NSCLC and esophageal cancer cohorts [60]. This rationale supports further investigation in histotype-specific experimental models. The safety profile was deemed acceptable, and the proposed Phase 2 dosage of tiragolumab was established.

Investigators have examined the humanized anti-TIGIT antibody vibostolimab (MK-7684) in clinical environments. In the original Phase 1 trial (MK-7684-001) involving human participants, vibostolimab was administered either as monotherapy or in conjunction with the PD-1 inhibitor pembrolizumab to patients with advanced solid tumors, including NSCLC. This study primarily assessed safety and tolerability while also examining preliminary signs of antitumor efficacy. Despite monotherapy exhibiting limited efficacy as a single agent, its combination with pembrolizumab was generally well tolerated and suggested immune activation and objective responses in select NSCLC patients, leading to the initiation of registrational phase III trials of dual TIGIT/PD-1 blockade [64]. These phase III trials, KeyVibe-003 and KeyVibe-007, subsequently met predetermined futility criteria for OS in 2025, and Merck discontinued the vibostolimab program in its entirety [63].

Recent clinical investigations have focused on domvanalimab (AB154), an Fc-silent anti-TIGIT antibody designed to block TIGIT without activating Fcγ receptors, so potentially reducing off-target effects. The EDGE-Gastric Phase 2 trial (NCT05329766) evaluated the efficacy of domvanalimab combined with the PD-1 inhibitor zimberelimab and chemotherapy in patients with previously untreated HER2-negative advanced gastric, gastroesophageal junction, or esophageal cancer. Simultaneous inhibition of TIGIT and PD-1, in conjunction with FOLFOX, demonstrated encouraging antitumor efficacy. The ORR was around 59%, the median progression-free survival (PFS) was roughly 12.9 months, and the median OS was 26.7 months for the entire cohort. The activity was equivalent to or greater in PD-L1–positive subgroups. The medication was well tolerated, and the immune-related adverse effects were consistent with the expected safety profile for combinations of checkpoint inhibitors. This data supports the continued evaluation of TIGIT/PD-1 inhibition in gastrointestinal malignancies [106].

Following the positive outcomes of Phase 2, Phase 3 studies of domvanalimab are underway. STAR-121 is a global randomized trial comparing domvanalimab plus zimberelimab with chemotherapy against pembrolizumab plus chemotherapy in first-line metastatic NSCLC. STAR-221 examines domvanalimab plus zimberelimab and chemotherapy versus nivolumab plus chemotherapy in gastric, gastroesophageal junction, and esophageal cancer. A recent independent data monitoring committee evaluated STAR-221 and recommended its cessation, as an interim study indicated it was ineffective in prolonging the lives of individuals with stomach and esophageal malignancies. This illustrates the difficulty of transforming initial promise into tangible therapeutic advantage. The extensive Phase 3 programs illustrate the complexity of immune checkpoint biology and the necessity for predictive biomarkers and optimal combination regimens [25].

Moreover, comprehensive research indicates that numerous anti-TIGIT antibodies, including ociperlimab, etigilimab, and various novel variants, are undergoing clinical trials for diverse cancer types and in conjunction with PD-1/PD-L1 inhibitors. Systematic analyses of clinical trial registries indicate over 70 registered anti-TIGIT trials, with around 47 actively recruiting individuals, several of which are in Phase 3, primarily targeting NSCLC, SCLC, and upper gastrointestinal malignancies. These diverse methodologies aim to discern tumor circumstances and patient subgroups that derive the greatest benefit from TIGIT-targeted immunotherapy [20].

**Table 5:** Clinical Trials Targeting TIGIT in Cancer Therapy.

Agent/Trial (NCT)	Cancer Type/Population	Phase & Design	Combination Partner(s)	Key Efficacy/Notes	Citations
Tiragolumab—CITYSCAPE	PD-L1–high metastatic NSCLC	Phase II, randomized	Atezolizumab (PD-L1)	Higher ORR and PFS vs. atezolizumab alone in PD-L1-selected NSCLC (37% vs. 21% in PD-L1-high subgroup); foundation for multiple phase III NSCLC trials	[20,58,107,108,109]
Tiragolumab—multiple NSCLC phase III	1L metastatic NSCLC (various PD-L1 strata)	Phase III, ongoing	Atezolizumab ± chemo	Early lung cancer studies positive; later phase III trials reported failures, tempering expectations	[7,20,45,108,109]
Vibostolimab (MK-7684)	Advanced solid tumors, NSCLC cohort	Phase I, dose-escalation/expansion	Pembrolizumab (PD-1)	In anti-PD-1–naïve NSCLC, vibostolimab + pembrolizumab ORR ≈ 26%; generally acceptable safety	[20,107,108,109]
Domvanalimab + Zimberelimab—LIVERTI (NCT05724563)	HCC refractory to prior anti-PD-1/L1	Phase II, single-arm	Zimberelimab (PD-1)	ORR 17.2%; median PFS 4.4 months; well-tolerated but primary endpoint not met	[67,83]
Etigilimab—Phase 1a/b	Metastatic/advanced solid tumors	Phase I, 3 + 3, mono and combo	Nivolumab (PD-1)	No DLTs up to 20 mg/kg; 1 partial response and prolonged stable disease; study stopped for business reasons	[20,70]
IBI939 (NCT04353830)	Advanced malignancies	Phase I, completed	Monotherapy	First-in-human safety and PK; primary endpoints AEs, DLTs	[110]
COM902 (NCT04354246)	Advanced cancers	Phase I, recruiting	Often with PD-1	Primary endpoints MTD and PK; ORR and CR as secondary	[13,87,110,111]
EOS-448 (NCT04335253)	Advanced cancers	Phase I/IIa, completed	Often combined with PD-(L)1 in later cohorts	RP2D and DLTs primary; developed as Fc-competent anti-TIGIT	[13,83,107,110]
HLX53 (NCT05394168)	Advanced/metastatic solid tumors or lymphoma	Phase I, not yet recruiting	Likely combinations after monotherapy run-in	Safety (MTD, DLTs) primary; ORR secondary	[13,87,110]
Multiple anti-TIGIT programs (≥5 in phase II+)	Mainly NSCLC; also GI, gynecologic, others	Phase II–III	Typically PD-1/PD-L1 co-blockade	>70 registered trials; signal strongest in PD-L1–positive NSCLC, but mixed late-phase results overall	[7,13,20,40,45,107,108,109,111,112]

NSCLC, non-small cell lung cancer; HCC, hepatocellular carcinoma; GI, gastrointestinal; PD-1, programmed cell death protein 1; PD-L1, programmed death-ligand 1; PFS, progression-free survival; ORR, objective response rate; DLTs, dose-limiting toxicities; PK, pharmacokinetics; AEs, adverse events; MTD, maximum tolerated dose; CR, complete response; RP2D, recommended phase II dose; Fc, fragment crystallizable region; 1L, first-line; chemo, chemotherapy; mono, monotherapy; combo, combination therapy; TIGIT, T-cell immunoreceptor with Ig and ITIM domains.

### 5.4. Combination Therapies with PD-1/PD-L1 Blockade

The principal rationale for combining TIGIT blockades with PD-1/PD-L1 inhibitors is that these checkpoints diminish anti-tumor immunity through distinct but convergent inhibitory pathways inside the TME. TIGIT is frequently observed in elevated levels on exhausted CD8^+^ T cells, NK cells, and intratumoral Tregs. It impedes effector effects and may inhibit the activating receptor CD226 by competing with ligands in the CD155/nectin axis. Simultaneously, PD-1 signaling inhibits T-cell receptor (TCR) signaling and costimulatory signaling on a broader scale. Prolonged antigen exposure and PD-L1 treatment may lead to “residual” inhibitory mechanisms, including the upregulation of TIGIT and other checkpoint molecules. Thus, dual inhibition is theoretically positioned to revitalize tired lymphocytes more efficiently than PD-L1 blocking alone, particularly in PD-L1-selected, immune-inflamed tumors where pre-existing antitumor immunity is apparent at baseline [60].

Synergy is hypothesized to arise from (i) restoring cytotoxic lymphocyte function, (ii) adjusting co-stimulation in favor of co-inhibition, and (iii) reorganizing suppressive cellular environments. Preclinical and translational evidence suggests that TIGIT inhibition can augment CD8^+^ T-cell proliferation and cytokine secretion, in addition to enhancing NK-cell-mediated tumor regulation. Simultaneously, PD-1/PD-L1 inhibition enhances TCR-proximal signaling and effector differentiation. These effects collectively enhance the probability that malfunctioning tumor-infiltrating lymphocytes (TILs) will recover sustained functionality. The extent of synergy may depend on the antibody format: specific anti-TIGIT molecules may utilize FcγR-dependent myeloid engagement and/or influence TIGIT^+^ Tregs, potentially modifying antigen presentation and local immunosuppression in a way that amplifies PD-L1 blockade. This “network” model lymphocyte reinvigoration coupled with myeloid/Treg remodeling has been validated by extensive studies linking clinical efficacy to macrophage and Treg attributes inside the tumor TME [40].

The crucial clinical indication that initiated significant TIGIT development stemmed from the CITYSCAPE study (tiragolumab + atezolizumab versus placebo + atezolizumab) in first-line, PD-L1-selected metastatic NSCLC. The preliminary report suggested that the combination of tiragolumab and atezolizumab improved the objective response rate (ORR) and PFS relative to atezolizumab monotherapy in the overall PD-L1-selected population (ORR 31.3% vs. 16.2%; median PFS 5.4 vs. 3.6 months; HR 0.57), with an even more pronounced benefit observed in the PD-L1-high (≥50%) subgroup (ORR 37% vs. 21%), thereby indicating biological additivity in an enriched population, notwithstanding the undeveloped OS data at that juncture [58].

Recent randomized data indicate that outcomes may vary based on the medicine and the setting In ARC-10 (Part 1), the Fc-silent anti-TIGIT antibody domvanalimab, in conjunction with the anti-PD-1 antibody zimberelimab, exhibited numerically enhanced outcomes in first-line PD-L1-high NSCLC compared to zimberelimab alone, including a median PFS of 11.5 months versus 6.2 months, with the median OS not yet reached at the time of analysis—results suggestive of additive efficacy in a PD-L1-high population that are being further evaluated in the ongoing phase III STAR-121 trial [68,113]. In contrast, a phase 3 adjuvant study of a vibostolimab–pembrolizumab coformulation in resected melanoma did not improve outcomes compared to pembrolizumab monotherapy, highlighting that TIGIT efficacy may be contingent upon specific contexts and disease biology, and may not be relevant in all PD-1–sensitive settings [114].

Combination strategies are being investigated beyond immunotherapy-naïve conditions to address acquired or primary resistance to PD-L1 inhibition. The phase 2 LIVERTI study of domvanalimab in conjunction with zimberelimab for immunotherapy-refractory hepatocellular cancer demonstrated a verified overall response rate (ORR) of 17.2% and a median PFS of 4.4 months. Correlative analyses suggested that ctDNA dynamics could function as a pharmacodynamic marker of response, reinforcing the hypothesis that TIGIT may serve as a compensatory checkpoint in specific PD-1–resistant scenarios, notwithstanding the limited magnitude of benefit and the possible requirement for biomarker-guided selection [67].

The concept of integrating TIGIT with PD-1/PD-L1 remains biologically intriguing. This occurs due to its mechanism of inhibiting exhausted lymphocytes and, in certain instances, altering the morphology of myeloid/Treg cells. However, the clinical record to date indicates that synergy is not consistently observed. The present trajectory of the field is shifting from a general application of “add TIGIT to PD-L1” towards mechanism-matched combinations and biomarker-enriched implementation, informed by tumor immunophenotype (inflamed vs. excluded), the integrity of the TIGIT/CD226/CD155 axis, and the dominance of suppressive niches (Treg/myeloid programs), while carefully considering antibody format and effector function.

## 6. Challenges of Anti-TIGIT Immunotherapy for Cancer

**Figure 14:**
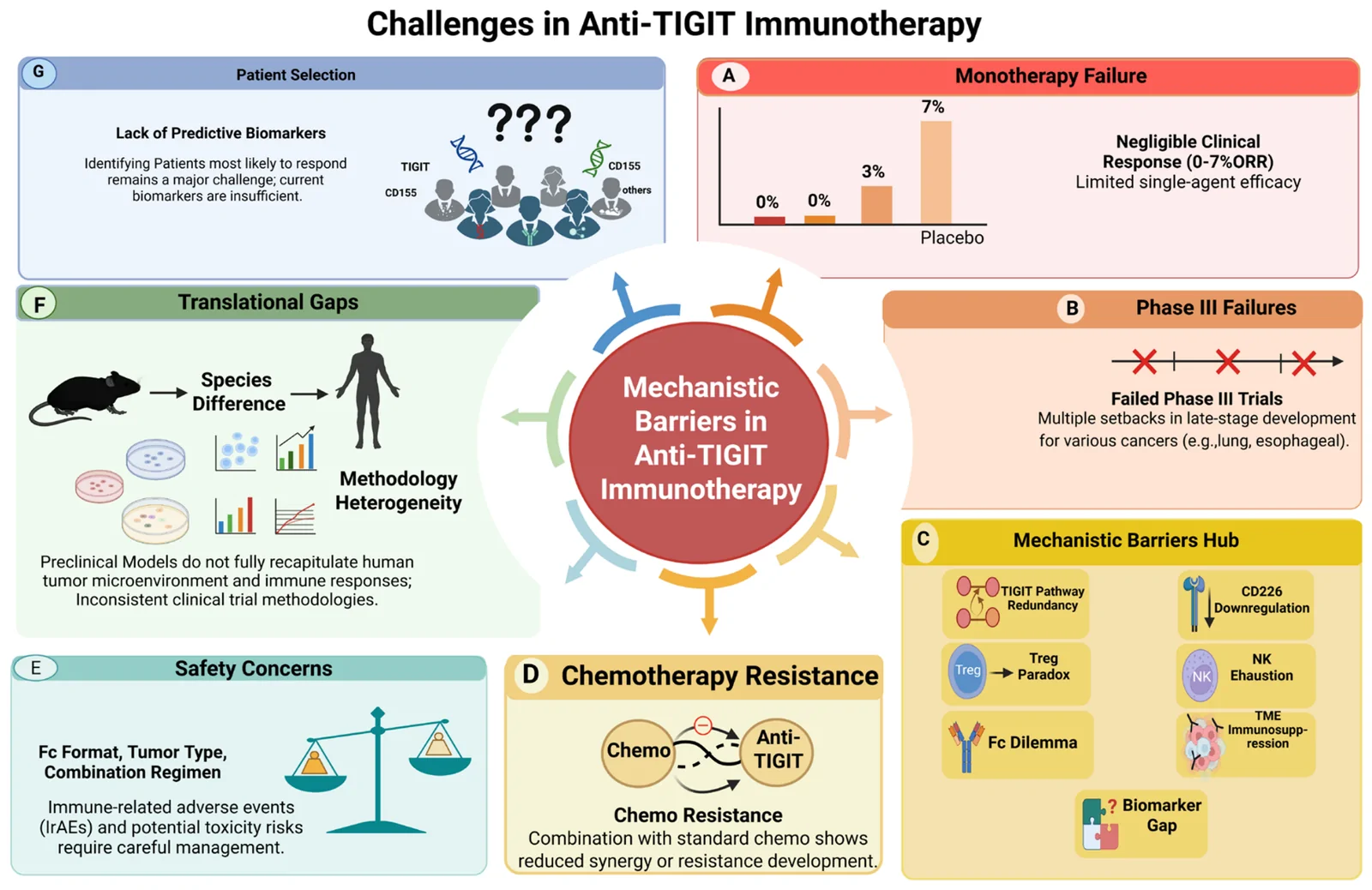
Seven Major Challenges in Anti-TIGIT Immunotherapy Development. Multi-panel figure presenting six categorized barriers: (**A**) monotherapy futility (ORR 0–7%); (**B**) near-universal Phase III trial failures (SKYSCRAPER-01, KEYVIBE-008); (**C**) seven mechanistic barriers including pathway redundancy, CD226 downregulation, Treg paradox, NK exhaustion, CD155 overexpression, myeloid infiltration, and absent biomarkers; (**D**) chemotherapy combination resistance; (**E**) safety heterogeneity by Fc format and tumor type; (**F**) translational gaps between murine models and human cancer biology. TIGIT; ORR, objective response rate; Treg, regulatory T cell; NK cells, natural killer cells; Fc, fragment crystallizable region; TME, tumor microenvironment; irAEs, immune-related adverse events. (**G**) represents absence of predictive biomarkers/patient-selection challenges. The red cross indicates therapeutic failure or lack of clinical benefit. Created in BioRender. Smail, S. (2026) https://BioRender.com/8j5gby3.

Anti-TIGIT immunotherapy has emerged as a promising immune checkpoint strategy in cancer treatment; however, multiple biological and clinical challenges continue to limit its overall therapeutic success. As illustrated in Figure 14, anti-TIGIT monotherapy generally demonstrates limited efficacy, with low objective response rates observed across several tumor types, indicating that TIGIT blockade alone is often insufficient to generate durable antitumor immunity. In addition, several late-stage clinical trials have failed to achieve their primary endpoints, highlighting the complexity of translating early preclinical success into clinical benefit. Mechanistically, resistance to anti-TIGIT therapy is influenced by multiple interconnected factors, including redundancy among inhibitory immune pathways, reduced CD226 co-stimulatory signaling, paradoxical effects on Tregs, persistent NK-cell exhaustion, high CD155 expression within the TME, immunosuppressive myeloid-cell infiltration, and the absence of reliable predictive biomarkers. Additional limitations arise from resistance to combination strategies involving chemotherapy and immune checkpoint inhibitors, as well as variability in toxicity and efficacy depending on antibody Fc design and tumor context. Importantly, significant translational gaps remain between murine tumor models and human cancers, limiting the predictive accuracy of preclinical findings and emphasizing the need for improved biomarker-driven and biologically informed therapeutic strategies.

### 6.1. Failure of Anti-TIGIT Monotherapy

Notwithstanding robust preclinical justification, anti-TIGIT monotherapy has persistently failed to exhibit significant clinical efficacy in human trials. In the phase I GO30103 trial, single-agent tiragolumab yielded no objective responses in patients with advanced solid tumors, although some tumor reduction was noted [60]. Vibostolimab monotherapy produced a 0% overall response rate in advanced solid tumors and just 3–7% in anti-PD-1/PD-L1-refractory non-small cell lung cancer [61,64]. The recurrent failures led to a 2025 study in Cancer Discovery stating that TIGIT inhibitors encountered significant setbacks following many phase III failures, resulting in major pharmaceutical corporations withdrawing from the field altogether [23]. Xuan and Chen (2025) presented an expansive theoretical framework for these failures, contending that the negative outcomes of TIGIT (alongside TIM-3 and ICOS) signify three core issues: the lack of tumor-specific immunosuppressive rationale in numerous checkpoint targets, the predominance rather than redundancy of immune evasion mechanisms within the TME, and the development of therapy-induced resistance [115]. Collectively, these findings demonstrate that TIGIT inhibition alone is inadequate to elicit clinically significant anti-tumor responses in human malignancies.

The strongest empirical lesson from the phase III program is that TIGIT expression, measured by immunohistochemistry (IHC), flow cytometry, or bulk RNA, is not by itself a useful patient-selection marker [109]. Retrospective TIGIT analyses across CITYSCAPE and the SKYSCRAPER trials failed to separate responders from non-responders in a way that would have supported prospective enrichment [40,58]. This is unsurprising because TIGIT expression scales with immune infiltration and antigen exposure: a “high-TIGIT” tumor could be a tumor with abundant exhausted effectors (potentially rescuable) or a tumor dominated by highly suppressive TIGIT^+^ Tregs (potentially aggravated by non-depleting blockade) [14,15,40,116]. The recent framework of Yang & Chen [115] independently argues that TIGIT lacks a tumor-specific immunosuppressive logic and that immune-evasion mechanisms in the TME are dominant rather than redundant, both of which imply that a single-marker biomarker approach is intrinsically limited. This same logic extends to disease selection: the divergent phase III outcomes described in Section 5.2., failure in SCLC and HCC against success in ESCC, track tumor-level CD155 expression and baseline immune context rather than TIGIT expression itself, reinforcing that a molecular biomarker framework and a disease-selection strategy are two views of the same underlying problem, not separate ones.

### 6.2. Variability in Clinical Responses and Phase III Clinical Trial Failures

Clinical responses to TIGIT-targeted immunotherapy vary considerably among patients because treatment efficacy depends on a complex interplay between tumor biology, immune cell composition, and the TME. Tumors with an immune-inflamed phenotype, characterized by abundant TILs and active IFN-γ signaling, generally respond more favorably to immune checkpoint inhibition than immune-desert or immune-excluded tumors [117]. Moreover, tumor heterogeneity can generate subclonal populations with distinct antigenic profiles, impaired antigen presentation, altered interferon signaling, and variable checkpoint expression, all of which contribute to immune escape and therapeutic resistance [118]. Patient-specific immunological factors, including genetic background, microbiome composition, and immune cell diversity, further influence responsiveness to TIGIT blockade, particularly because TIGIT is expressed on multiple immune populations, including exhausted CD8^+^ T cells, NK cells, and Tregs [119].

The immunosuppressive architecture of TME also represents a major obstacle to successful TIGIT-targeted therapy. Tumor-associated macrophages (TAMs), myeloid-derived suppressor cells (MDSCs), stromal elements, and suppressive cytokines can inhibit effector T-cell function and limit durable antitumor immunity even when TIGIT signaling is successfully blocked [120]. Consequently, TIGIT inhibition alone may be insufficient in many tumors where multiple suppressive mechanisms coexist. These observations highlight the need for improved biomarker-driven patient selection and comprehensive immune profiling to identify patients most likely to benefit from TIGIT-based treatment and to guide rational combination strategies that overcome resistance mechanisms within the TME [118,119,120].

The biological complexity underlying TIGIT signaling is reflected in the disappointing outcomes of most phase III clinical trials. The SKYSCRAPER-02 trial in extensive-stage small-cell lung cancer and the SKYSCRAPER-01 trial in PD-L1-high non-small-cell lung cancer failed to demonstrate meaningful improvements in OS despite promising earlier studies [22,26,121]. Similarly, SKYSCRAPER-03, SKYSCRAPER-14/IMbrave152, STAR-221, AdvanTIG-302, and the KeyVibe program all failed to meet primary efficacy endpoints, leading several pharmaceutical companies to discontinue anti-TIGIT development programs [24,25,26,63,71,122]. The notable exception was SKYSCRAPER-08 in esophageal squamous cell carcinoma, which demonstrated significant improvements in both progression-free and OS, suggesting that TIGIT blockade may be effective only in selected biological contexts [123]. Collectively, these results indicate that future success with anti-TIGIT therapies will likely depend on disease-specific biology, biomarker-guided patient selection, and a better understanding of the immune context in which the TIGIT–CD155 axis plays a dominant role [22,24,25,26,63,71,121,122].

Importantly, the positive outcome of SKYSCRAPER-08 should not be interpreted as reversing the broader clinical experience of the TIGIT field. Despite this encouraging result in ESCC, the overwhelming majority of phase III anti-TIGIT programs across NSCLC, SCLC, HCC, melanoma, and gastric cancer have failed to demonstrate clinically meaningful improvements in survival outcomes or meet their primary endpoints. Therefore, SKYSCRAPER-08 is best viewed as evidence that TIGIT blockade may be effective in selected biological contexts rather than validation of TIGIT inhibition as a universally applicable immunotherapeutic strategy. The contrast between ESCC and multiple negative phase III studies further supports the importance of disease-specific biology, biomarker-guided patient selection, and context-dependent dependence on the TIGIT–CD226–CD155 axis [21,22,24,25,26,122].

#### Trial Design, Chemotherapy Backbone, and Statistical Considerations in Phase III Failures

The disappointing outcomes of multiple phase III anti-TIGIT programs likely reflect not only biological limitations of the TIGIT pathway but also challenges related to trial design, chemotherapy backbone selection, and statistical assumptions. Most registrational studies were launched shortly after the positive phase II CITYSCAPE trial and generally adopted broad combination strategies in which anti-TIGIT antibodies were added to an established PD-1/PD-L1-based standard of care without prospective biomarker enrichment beyond PD-L1 expression [21,58]. Consequently, patient populations remained biologically heterogeneous, potentially diluting treatment effects within subgroups most likely to benefit from TIGIT blockade [45,109].

Trial design heterogeneity may also have contributed to divergent outcomes across disease settings. CITYSCAPE enrolled a relatively small PD-L1-selected NSCLC population and demonstrated a promising signal of activity, whereas subsequent phase III trials evaluated substantially larger and more diverse populations, including SCLC, NSCLC, HCC, and ESCC, each characterized by distinct immune microenvironments and different levels of dependence on the TIGIT–CD226–CD155 axis [21,22,26,58]. The positive results observed in SKYSCRAPER-08, compared with the negative outcomes of SKYSCRAPER-02 and SKYSCRAPER-14, suggest that tumor-specific biology may be more important than the checkpoint target itself and emphasize the need for disease-specific development strategies rather than broad pan-tumor deployment of anti-TIGIT antibodies [22,26].

The choice of chemotherapy backbone may represent an additional confounding factor. Cytotoxic chemotherapy can enhance immunotherapy efficacy through immunogenic cell death, increased antigen release, and improved antigen presentation. However, chemotherapy may also cause lymphodepletion and impair the functionality of the same effector T-cell populations that TIGIT blockade is intended to reinvigorate [22,121]. Consequently, the interaction between anti-TIGIT therapy and chemotherapy is unlikely to be uniformly beneficial across all tumor types. The failure of tiragolumab combined with atezolizumab and carboplatin-etoposide in SCLC contrasts with the success of tiragolumab plus chemotherapy in ESCC and indicates that the immunological effects of chemotherapy are highly context dependent [22,26].

Statistical considerations should also be taken into account when interpreting anti-TIGIT trial outcomes. Several phase III programs were designed based on effect sizes extrapolated from the relatively small CITYSCAPE study, raising the possibility that the magnitude of benefit observed in early-phase development overestimated the true treatment effect. In addition, anti-TIGIT therapy likely benefits only a biologically defined subgroup of patients, whereas most phase III trials enrolled unselected or broadly PD-L1-selected populations. Under such circumstances, clinically meaningful benefit in a minority subgroup may be diluted when analyzed across the entire intention-to-treat population, resulting in negative primary analyses despite activity in specific biological niches [21,40,58,109]. These observations support future enrichment strategies incorporating CD226 status, CD155 expression, immune-context signatures, and spatial TME profiling to improve statistical power and enhance the probability of detecting treatment effects in responsive populations [40,124,125,126].

### 6.3. Mechanistic Challenges

The clinical shortcomings of anti-TIGIT therapy arise from various interconnected mechanistic obstacles that collectively explain the inconsistency in translating preclinical promise into therapeutic advantage. Collectively, current evidence suggests that not all proposed resistance mechanisms contribute equally to anti-TIGIT clinical failure. Among the various hypotheses, loss of CD226 functionality, inadequate biomarker-guided patient selection, checkpoint redundancy, and disease-specific immune context are supported by the strongest combination of mechanistic, translational, and clinical evidence. In contrast, the relative contribution of Fc-dependent effects, NK-cell fratricide, and several emerging resistance pathways remains less certain and requires prospective clinical validation. Consequently, future development efforts should prioritize restoration of CD226 signaling, biomarker-enriched trial design, and disease-specific patient selection rather than assuming universal applicability of TIGIT blockade across tumor types.

#### 6.3.1. Incomplete Mechanistic Understanding and Functional Redundancy with PD-1

Ding et al. (2025) [127] contended that the clinical ineffectiveness of TIGIT inhibitors stemmed from an inadequate mechanistic comprehension, an absence of predictive biomarkers, and functional redundancy with PD-1. They asserted that future success necessitates an emphasis on PD-1-refractory tumors, enhanced humanized preclinical models, and collaboratively developed companion diagnostics, as opposed to the expedited commercial development that typified the initial phase of the field [127]. This functional redundancy indicates that in several tumor situations, TIGIT blockage does not yield additional immunological advantages beyond those previously attained through PD-1 blocking, as both pathways converge on the identical downstream costimulatory receptor CD226.

#### 6.3.2. The Fc Region Dilemma

The selection of IgG isotype and Fc gamma receptor interaction has become a pivotal and unsolved factor in the production of anti-TIGIT antibodies. A pivotal 2024 study published in Nature by Guan et al. revealed that tiragolumab surrogate antibodies in an Fc-competent IgG1 format activated tumor-associated macrophages, monocytes, and dendritic cells via Fc gamma receptor interaction, facilitating the transition of anti-tumor CD8^+^ T cells from an exhausted effector-like state to a more memory-like state. Also, a high baseline of intratumoral macrophages and Tregs correlated with improved outcomes in patients receiving tiragolumab in conjunction with atezolizumab, but not with atezolizumab alone [40]. A 2025 review in Cancer Immunology and Immunotherapy defined the paradox wherein the Fc-competent format, which activates myeloid cells and depletes Tregs, also poses the risk of annihilating exhausted effector T cells instead of activating them. This engenders a fundamental tension between immune activation and immune cell depletion, potentially elucidating the deterioration observed in certain patients undergoing therapy [83].

The Fc region dilemma transcends T cells, highlighting a hitherto overlooked issue in NK cell biology. Due to the strong co-expression of CD16, the Fc receptor responsible for mediating antibody-dependent cellular cytotoxicity (ADCC), and TIGIT on activated NK cells, ADCC-capable anti-TIGIT antibodies can induce NK cell fratricide, wherein TIGIT-expressing NK cells are eliminated by other CD16-expressing NK cells. Hasan et al. (2023) demonstrated at American Association for Cancer Research (AACR) that TIGIT knockout in ex vivo-expanded primary human NK cells inhibited fratricide while concurrently augmenting antitumor cytotoxicity, upregulating mechanistic Target Of Rapamycin Complex 1 (mTORC1) signaling, elevating the basal glycolytic rate, and enhancing degranulation, indicating that engineered NK cell strategies could synergize with anti-TIGIT antibody therapy by circumventing this self-destructive cycle [128]. Additionally, clinical data from a phase II study of the Fc-silent anti-TIGIT antibody domvanalimab combined with zimberelimab in anti-PD-1-refractory hepatocellular carcinoma revealed a 17.2% confirmed overall response rate, including one complete response among 29 patients. The investigators concluded that an intact Fc domain may not be necessary for anti-TIGIT antibodies to elicit antitumor effects, thereby directly contesting the assumption that Fc-mediated effector functions are crucial for clinical efficacy [67].

The collective evidence suggests that the central question is not whether Fc-active or Fc-silent anti-TIGIT antibodies are universally superior, but rather which immune context favors each mechanism. Fc-active antibodies may provide therapeutic benefit through depletion of TIGIT^+^ Tregs and activation of FcγR-expressing myeloid cells, thereby remodeling an immunosuppressive TME [40,83,84]. However, because TIGIT is also expressed on exhausted but potentially recoverable CD8^+^ T cells and NK cells, Fc-mediated effector functions may simultaneously eliminate cellular populations required for antitumor immunity [83,128]. Conversely, Fc-silent antibodies preserve TIGIT-expressing effector lymphocytes but may sacrifice potentially beneficial Treg depletion and myeloid reprogramming [67,129]. Consequently, the unresolved Fc dilemma is best viewed as a context-dependent balance between suppressor-cell depletion and effector-cell preservation, rather than a simple choice between two antibody formats. Future studies should determine whether immune composition, including the relative abundance of TIGIT^+^ Tregs, macrophages, CD8^+^ T cells, and NK cells, can guide optimal Fc-format selection [40,67,83].

#### 6.3.3. CD226 Downregulation

For mechanistic clarity, resistance to anti-TIGIT therapy can be broadly divided into TIGIT-axis-specific mechanisms and generic checkpoint-resistance mechanisms. TIGIT-specific mechanisms are directly linked to dysfunction of the TIGIT-CD155-CD226 pathway and include CD226 downregulation or degradation, persistent CD155-mediated suppression, an unfavorable TIGIT/CD226 balance within intratumoral Tregs, and compensatory signaling through alternative CD155/nectin family receptors such as CD112R and CD96 [9,30,67,116,129,130,131]. These mechanisms directly reduce the biological effectiveness of TIGIT blockade and may explain treatment failure even in tumors with high TIGIT expression.

In contrast, generic checkpoint-resistance mechanisms are not unique to TIGIT inhibition and are shared across multiple immunotherapeutic platforms. These include alternative checkpoint upregulation (PD-1, LAG-3, TIM-3, CTLA-4), defective antigen presentation, loss of MHC class I expression, immunosuppressive TAM and MDSC infiltration, stromal exclusion, and low tumor mutational burden [59,110,120,128]. Although these mechanisms may contribute substantially to anti-TIGIT failure, they are not directly caused by dysfunction of the TIGIT-CD155-CD226 axis itself and often require additional therapeutic strategies beyond TIGIT blockade alone.

TIGIT blockade functions chiefly by reinstating signaling via its costimulatory homologue CD226, which competes with TIGIT for the same ligands CD155 and CD112. However, CD8^+^ tumor-infiltrating lymphocytes in melanoma and other solid tumors gradually diminish CD226 expression, which significantly restricts the effectiveness of TIGIT inhibition due to the absence of a costimulatory receptor to “activate.” [132]. Banta et al. (2022) [19] conducted a pivotal mechanistic investigation in Immunity, revealing that PD-1 impedes the phosphorylation of CD226 and CD28 through its ITIM-containing intracellular domain, whereas TIGIT constrains CD226 costimulation through an alternative mechanism by obstructing its interaction with the shared ligand CD155. Consequently, complete restoration of CD226 signaling and optimum anti-tumor CD8^+^ T cell responses necessitate the concurrent inhibition of both TIGIT and PD-1, offering the fundamental molecular justification for combinatorial targeting in clinical settings [19].

TIGIT blockade does not directly activate T cells; rather, it functions by removing inhibitory pressure on the competing co-stimulatory receptor CD226. Therefore, the biological activity of anti-TIGIT therapy is fundamentally dependent on the presence of functional CD226 signaling. During chronic antigen exposure, tumor-reactive CD8^+^ T cells progressively lose surface CD226, while sustained CD155 expression in the TME promotes CD226 internalization, degradation, and signaling dysfunction [130]. As a result, exhausted T cells may remain TIGIT-high but become CD226-low or CD226-negative, effectively eliminating the co-stimulatory pathway that anti-TIGIT antibodies are designed to restore. Under these conditions, blocking TIGIT removes an inhibitory receptor but fails to generate productive activation because the corresponding activating pathway is no longer available. Mechanistically, this creates a state in which TIGIT expression is present, yet TIGIT dependence is absent. This concept may explain the disconnect between high TIGIT expression and poor clinical responsiveness observed across several anti-TIGIT studies and suggests that CD226 status is a more biologically relevant determinant of treatment efficacy than TIGIT expression alone [9,19,130,131].

Recent translational studies further support this model. Braun et al. demonstrated that tumor-derived CD155 induces phosphorylation-dependent internalization and degradation of CD226 in CD8^+^ T cells, thereby reducing the capacity of TIGIT blockade to restore antitumor immunity [130]. Similarly, Jin et al. showed that anti-TIGIT therapy is effective primarily in CD226-high CD8^+^ T cells because restoration of antitumor function requires intact CD226 tyrosine phosphorylation and downstream signaling [131]. Collectively, these findings suggest that the major biological limitation of TIGIT inhibition is not necessarily insufficient TIGIT expression, but rather progressive erosion of the CD226 signaling axis during T-cell exhaustion. Consequently, future anti-TIGIT strategies may require either prospective selection of CD226-positive patients or therapeutic approaches capable of restoring CD226 expression and function before TIGIT blockade can achieve meaningful clinical activity [19,130,131,133].

#### 6.3.4. Upregulation of Alternative Immune Checkpoints

Tumors can counteract TIGIT blockade by upregulating alternative inhibitory receptors, including PD-1, TIM-3, LAG-3, and CTLA-4. The emergence of these alternative checkpoints as resistance mechanisms has been identified as a significant limitation of TIGIT-targeted therapy [59]. CD8^+^ T cells specific to circulating tumor antigens and CD8^+^ tumor-infiltrating lymphocytes that co-express PD-1, TIGIT, and TIM-3 demonstrate differing degrees of T-cell dysfunction, and inhibiting any single checkpoint may not adequately restore effector function due to this multi-checkpoint co-expression [132].

#### 6.3.5. The Immunosuppressive Tumor Microenvironment

Immunologically “cold” tumors present a significant obstacle to anti-TIGIT therapy. These tumors have a deficiency of effector T cells in the TME, a modest mutational load, a little neoantigen burden, and an immunosuppressive environment predominated by inhibitory cell populations [134]. The principal immunosuppressive cellular elements comprise tumor-associated macrophages polarized to the M2 phenotype, MDSCs, Tregs, and cancer-associated fibroblasts, all of which facilitate the establishment of an environment that inherently opposes checkpoint blockade-mediated immune activation [120,135].

#### 6.3.6. TIGIT/CD155 Axis Mediates Acquired Resistance

Resistance mechanisms are worth separating into TIGIT-CD155-CD226-specific and generic checkpoint-failure categories, since prior reviews conflate the two and thereby overestimate what any TIGIT-specific intervention can deliver. Pathway-specific mechanisms include: CD226 downregulation or shedding on exhausted TIL, removing the receptor TIGIT blockade is designed to liberate [130,131,132]; a high TIGIT/CD226 ratio in intratumoral Tregs that sustains suppression despite receptor blockade [116,136]; compensatory CD112R signaling via CD112, a parallel inhibitory input not neutralized by anti-TIGIT antibodies [30,137]; and tumor CD155 heterogeneity, which blunts pharmacodynamic effect where expression is low [124,138]. Generic checkpoint-resistance mechanisms include alternative checkpoint upregulation (PD-1, LAG-3, TIM-3, CTLA-4) [59,110], loss of MHC class I/antigen-presentation machinery, MDSC and M2-macrophage dominance in cold tumors [120,135], and low tumor mutational burden. TIGIT-PD-1 co-blockade addresses only the first generic mechanism; the rest require orthogonal interventions such as tumor-priming or myeloid reprogramming [139].

Kawashima et al. (2021) [138] established in the Journal for Immunotherapy of Cancer that the TIGIT/CD155 axis directly facilitates resistance to PD-1 and CTLA-4 checkpoint inhibition in melanoma patients with an inflammatory TME. Their research shown that CD155 expression elevated in surviving tumor cells following co-culture with tumor-infiltrating lymphocytes from responsive patients, subsequently inhibiting TIGIT-positive T-cell activation and creating a feedback loop of acquired resistance [138]. This discovery is significant as it indicates that in individuals with inflammatory tumors who initially respond to immunotherapy, the TIGIT/CD155 axis may develop as a mechanism of secondary resistance.

#### 6.3.7. Patient Selection and Biomarker Absence

Currently, there are no approved companion diagnostics for anti-TIGIT therapy, and this lack of predictive biomarkers is well acknowledged as a significant obstacle to clinical success. Key factors influencing the outcome of clinical programs, such as antibody origin, IgG isotype, Fc gamma receptor affinity, and dosing regimen, are not fully optimized. Additionally, determining which patients will derive benefit from TIGIT blockade remains a major unresolved issue in the field [109].

### 6.4. Challenges Specific to Combination with Chemotherapy

The SKYSCRAPER-02 failure in SCLC illustrated that the addition of anti-TIGIT to chemotherapy and anti-PD-L1 does not consistently enhance results, even when all three treatments are administered concurrently from the beginning [22]. Chemotherapy can cause lymphodepletion, which may negate the T cell activation anticipated from TIGIT blockade. Additionally, specific tumor types, such as SCLC, may possess inherent biological traits such as a low neoantigen burden and a neuroendocrine phenotype that make them fundamentally resistant to checkpoint-based immune activation, irrespective of the number of targeted pathways. [121]. Nonetheless, the efficacy of SKYSCRAPER-08 in esophageal squamous cell carcinoma illustrates that the chemotherapy-immunotherapy-anti-TIGIT triple combination can yield statistically significant and clinically relevant enhancements in both PFS and OS when tumor biology is advantageous, indicating that the difficulty resides not in the combination concept but in the judicious selection of tumor types [26].

### 6.5. Mechanisms of Resistance and Non-Responsiveness

Resistance and insufficient response to TIGIT-targeted immunotherapy arise because TIGIT operates not as a solitary inhibitory receptor, but as part of a more extensive, highly adaptable immune-suppressive network. In several malignancies, the absence of response is ascribed to a confluence of low tumor immunogenicity, inadequate antigen presentation, insufficient T-cell infiltration, and dominant suppressive stromal and regulatory cell mechanisms. These qualities create a context in which the exclusive inhibition of TIGIT may be biologically inadequate, especially when effector cells are either scarce or overly defective to be rehabilitated. This explains why anti-TIGIT therapies that amplify initial signals have not demonstrated consistent efficacy across various cancer types and treatment scenarios, and why tumors that do not respond are typically non-responsive due to the intricate tumor-immune ecosystem, rather than merely TIGIT expression [140].

A crucial component of this is adaptive feedback in exhausted T cells, specifically through the TIGIT–PD-1–CD226 signaling pathway. Research indicates that TIGIT and PD-1 inhibit antitumor CD8^+^ T-cell functionality by different yet converging processes that eventually compromise CD226, an essential costimulatory receptor necessary for successful T-cell activation. Extended exposure to antigens may lead to the ineffectiveness of suppressing a singular inhibitory pathway, thereby preserving malfunctioning T cells in a state of partial exhaustion instead of totally revitalizing them. In this context, the apparent primary resistance to TIGIT monotherapy may arise from the redundant circuitry of the inhibitory program, necessitating the simultaneous alleviation of several checkpoint constraints to restore effective cytotoxicity, rather than merely inhibiting TIGIT [19].

A secondary mechanism involves the compensatory upregulation of checkpoints, whereby tumors and chronically activated immune cells shift to alternative inhibitory receptors in response to treatment pressure. Exhausted T and NK cells often co-express TIGIT with PD-1, LAG-3, TIM-3, and other checkpoint markers, indicating a sophisticated inhibitory regulation of proliferation, cytokine production, and cytolytic function. Thus, despite the suppression of TIGIT, the compensatory dominance of LAG-3, TIM-3, or other pathways may sustain immune paralysis. The flexibility of TIGIT is a principal rationale for its optimal use in judicious combinations rather than as a standalone strategy, indicating that TIGIT expression alone is insufficient as a biomarker for predicting response [110].

A major impediment is the immunosuppressive regulatory T-cell population, especially intratumoral Tregs with a significant TIGIT/CD226 imbalance. Research on human melanoma reveals that TIGIT-positive Tregs are significantly suppressive, enduring, and abundant in tumors, but CD226 diminishes Treg suppressive function. An elevated TIGIT/CD226 ratio in tumor-infiltrating Tregs was substantially correlated with heightened Treg prevalence and unfavorable clinical outcomes after immune checkpoint suppression. This discovery is relevant to TIGIT resistance as it suggests that, in specific patients, TIGIT functions not only as a marker for exhausted effector cells that can be revitalized but also as a sign of a significantly suppressive Treg state that actively promotes immunological evasion. In specific tumors, simple receptor blockade may be inadequate unless the therapy also reduces, depletes, or reprograms the suppressive Treg populations [116].

The extensive immunosuppressive myeloid and stromal milieu also perpetuates resistance. Recent mechanistic and translational research suggests that the efficacy of anti-TIGIT may rely on Fc receptor interaction and the subsequent reconfiguration of tumor-associated macrophages, monocytes, dendritic cells, and Treg-enriched microenvironments. This is noteworthy as it suggests the opposite: if these suppressive myeloid circuits persist, blocking TIGIT may be inadequate to convert the TME into a state favorable for sustained T-cell regulation. A lack of response may suggest that the lymphocytes are exhausted and that the adjacent suppressive environment has not been adequately altered. The observations indicate that variations in anti-TIGIT antibodies, particularly regarding Fc functioning, may influence the inconsistent efficacy found in studies, so partially elucidating why certain treatments exhibit mechanistic potential yet provide limited clinical advantages [45].

### 6.6. Safety and Adverse Effects

The safety profile of TIGIT-targeted treatment has thus far been predominantly acceptable and equivalent to that of traditional ICIs. This is especially true when anti-TIGIT agents are used in conjunction with PD-1/PD-L1 blockade instead of within highly intensified immunotherapy regimens. Preliminary clinical studies of tiragolumab, an extensively studied anti-TIGIT antibody, demonstrated its efficacy as both a monotherapy and in conjunction with atezolizumab. Adverse events were predominantly aligned with those anticipated from checkpoint inhibition, with no distinct toxicity pattern associated with TIGIT blocking emerging. This observation is significant as it indicates that the primary safety concern related to TIGIT-directed therapy is not the introduction of a new class-specific toxicity, but rather the potential increase in the frequency or severity of existing irAEs when administered alongside current PD-1/PD-L1-based treatments [60].

Randomized data indicate that the primary side effects of anti-TIGIT combinations encompass fatigue, pruritus, rash, infusion-related reactions, pyrexia, and laboratory abnormalities, alongside the usual immune-mediated toxicities linked to checkpoint inhibitors, including hepatitis, colitis, pneumonitis, thyroid dysfunction, adrenal insufficiency, and dermatitis. The phase 2 CITYSCAPE trial of tiragolumab combined with atezolizumab for first-line PD-L1-positive non-small cell lung cancer showed enhanced efficacy, although it also led to clinically significant treatment-related toxicity. The primary grade 3 or higher treatment-related adverse event was an increase in lipase levels, and there were two treatment-related fatalities in the tiragolumab cohort, one attributable to pyrexia and the other to infection. The events do not exhibit a clear pattern of toxicity for TIGIT; yet, they suggest that incorporating TIGIT blocking with PD-L1 inhibition may still lead to serious and often lethal side consequences. This signifies that meticulous supervision is crucial in standard procedures and upcoming experiments [58].

Safety may depend on the TME, additional therapy, and antibody composition, particularly whether the anti-TIGIT antibody exhibits Fc-enabled or Fc-silent properties. In hepatocellular carcinoma, the incorporation of tiragolumab with atezolizumab and bevacizumab did not markedly increase treatment-related or immune-mediated adverse effects, and researchers did not detect any major new safety issues. This suggests that TIGIT combinations can sometimes preserve a suitable therapeutic index, even in multi-drug regimens. The wider context of therapeutic progress suggests that tolerance cannot be presumed in all endeavors. For example, certain late-stage anti-TIGIT medicines have faced challenges, and at least one notable vibostolimab trial for lung cancer was discontinued due to reports of a considerable occurrence of adverse effects. The evidence indicates that the safety of TIGIT inhibition is context-dependent, requiring a specific risk assessment for each molecule and regimen instead of a generalized evaluation for the entire class [141,142].

In medical practice, the management of irAEs linked to TIGIT-based therapy follows the same principles as those for other checkpoint inhibitors, as no specific management approach for TIGIT has been established to far. Timely identification, evaluation of severity, temporary discontinuation of treatment for clinically significant toxicity, and prompt administration of corticosteroids for moderate to severe immune-mediated reactions are essential. Organ-specific escalation strategies are essential; for example, suspected pneumonitis, hepatitis, myocarditis, neurologic toxicity, or severe colitis require prompt assessment and often necessitate subspecialty intervention, while steroid-refractory cases may demand second-line immunosuppression, including infliximab, vedolizumab, mycophenolate mofetil, or other targeted therapies depending on the affected organ. The existing ASCO and SITC guidelines emphasize the significance of patient education, initial laboratory assessment, ongoing monitoring during treatment, and multidisciplinary management to reduce morbidity and avert delayed diagnosis. Therefore, as TIGIT combinations advance, the most judicious implementation strategy includes not only pharmacovigilance during trials but also the rigorous application of established frameworks for managing irAEs linked to PD-1, PD-L1, and CTLA-4 inhibitors [142].

### 6.7. Translational and Methodological Challenges

A notable translational challenge in the TIGIT domain is the discrepancy between preclinical promise and inconsistent clinical effectiveness. The initial rationale for TIGIT blockade was predominantly based on syngeneic mouse models and limited functional assays; however, these models fail to accurately reflect the complexities of human tumors, especially the chronic immune editing, prior treatment exposure, and spatially heterogeneous suppressive niches found in patients with advanced cancer. Furthermore, the molecular pathways under consideration are not consistent across species: the importance of the TIGIT–CD226–CD155 axis, the role of NK cells in contrast to exhausted T cells, and the impact of Fc receptor engagement may differ between murine models and human tumors. This explains why strong anticancer activity in experimental animals has not consistently led to success in phase 3 trials and why molecular conclusions derived from a specific antibody platform cannot be uniformly applied to the entire class [45].

#### 6.7.1. Species Differences Between Murine Models and Human Disease

Three species differences complicate translation: CD226 is generally preserved on murine CD8^+^ TIL during tumor progression but progressively lost on exhausted human TIL [130,131,132]—the very population TIGIT blockade is meant to rescue; the murine FcγR repertoire differs quantitatively from the human one, so Fc-dependent effector functions shown in mouse do not translate directly [129,143]; and NK-cell CD16 co-expression with TIGIT, and the resulting fratricide risk from Fc-competent antibodies, differs between species [128]. These differences argue for humanized mouse models incorporating human FcγRs and CD226-low TIL as a minimum standard for de-risking new candidates.

#### 6.7.2. Limitations of Current Tumor Models

Standard syngeneic models (CT26, MC38, B16, Renca) are inbred, treatment-naïve tumors that grow rapidly in immunocompetent hosts. They do not reproduce the chronic immune editing, prior treatment exposure, spatial heterogeneity, or suppressive-niche architecture of human advanced cancer, and systematically overestimate checkpoint-blockade effect sizes [144,145]. GEMM and humanized-mouse platforms narrow but do not close this gap—the TIGIT preclinical-to-phase-III mismatch is partly a model-system problem, not only a target-biology one. Future preclinical work should prioritize human FcγR knock-in mice, tumors with induced CD226-low TIL, prior anti-PD-1 exposure to model the second-line population TIGIT is now most likely to be studied in, and physiologic (not overexpressed) CD155 levels [115,124,130,131,135].

Taken together, available evidence suggests that the widespread failure of anti-TIGIT therapies should not be interpreted as evidence of poor target biology alone. Instead, the strongest support currently exists for a multifactorial model in which inadequate biomarker-guided patient selection, Tumor-type-specific dependence on the TIGIT-CD155-CD226 axis, CD226 dysfunction, and checkpoint network redundancy collectively account for much of the observed clinical failure [9,19,40,130,131]. By comparison, the relative contribution of antibody-format selection, Fc-dependent mechanisms, and several emerging resistance pathways remains less certain and requires prospective validation [40,83,129,143]. Similarly, the contrast between the positive SKYSCRAPER-08 trial and multiple negative studies in NSCLC, SCLC, HCC, melanoma, and gastric cancer suggests that disease context may be a more important determinant of success than TIGIT expression alone [21,22,25,26,122]. Consequently, current evidence favors a model of context-dependent therapeutic failure rather than a simple conclusion that TIGIT is an invalid immunotherapeutic target.

## 7. Proposed Solutions

**Figure 15:**
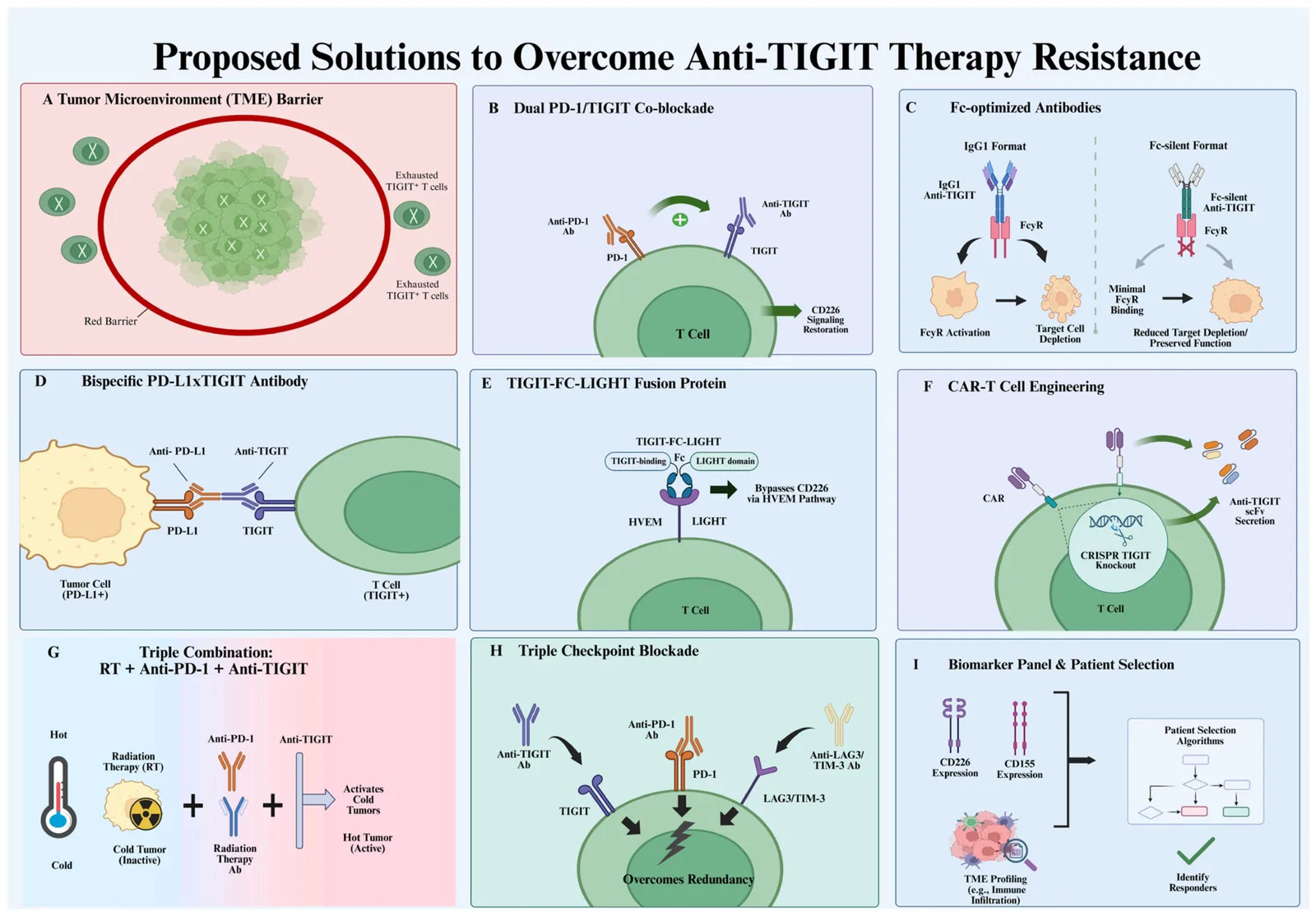
Proposed Solutions to Overcome Anti-TIGIT Therapy Resistance. Nine-panel (**A**–**I**) solution map: (**A**) TME resistance landscape; (**B**) dual PD-1/TIGIT co-blockade restoring CD226 signaling; (**C**) Fc-optimized antibodies (GASDALIE/Fc-silent); (**D**) bispecific PD-L1 × TIGIT antibodies; (**E**) TIGIT-Fc-LIGHT fusion bypassing CD226 via HVEM; (**F**) CAR-T TIGIT knockout or scFv secretion; (**G**) triple combo (RT + anti-PD-1 + anti-TIGIT) converting cold tumors; (**H**) triple checkpoint blockade (TIGIT + PD-1 + LAG-3/TIM-3); (**I**) biomarker-guided patient selection using CD226, CD155, TIL, and IFN-γ profiling. TIGIT, T cell immunoreceptor with Ig and ITIM domains; TME, tumor microenvironment; PD-1, programmed cell death protein 1; PD-L1, Fc, fragment crystallizable region; FcγR, Fc gamma receptor; HVEM, herpesvirus entry mediator; CAR-T, chimeric antigen receptor T-cell; CRISPR, clustered regularly interspaced short palindromic repeats; scFv, single-chain variable fragment; RT, radiotherapy; LAG-3, lymphocyte activation gene-3; TIM-3, T-cell immunoglobulin and mucin-domain containing-3. Created in BioRender. Smail, S. (2026) https://BioRender.com/b4fanvf.

As illustrated in Figure 15, combined blockade of TIGIT with PD-1 or other inhibitory checkpoints such as LAG-3 and TIM-3 may restore exhausted T-cell activity and enhance CD226-mediated immune stimulation. Novel therapeutic designs, including Fc-engineered antibodies, bispecific PD-L1 × TIGIT antibodies, and TIGIT-Fc-LIGHT fusion proteins, aim to strengthen immune activation within the TME. In addition, TIGIT-knockout CAR-T cells and radiotherapy-based triple-combination therapies may help convert immunologically “cold” tumors into more responsive tumors. Finally, biomarker-guided patient selection using CD226, CD155, tumor-infiltrating lymphocytes, and IFN-γ signatures may improve treatment stratification and clinical outcomes.

### 7.1. Dual PD-1/PD-L1 and TIGIT Co-Blockade

The dual co-blockade of PD-1/PD-L1 and TIGIT is the best recognized and mechanistically sound combo technique. The justification stems from the convergence of two inhibitory pathways on CD226 signaling: as PD-1 and TIGIT inhibit CD226 via distinct mechanisms, concurrent blockade of both receptors is necessary for complete restoration of costimulatory signaling and maximal anti-tumor CD8^+^ T cell responses [19]. Preclinical models frequently exhibit synergy between TIGIT and PD-1/PD-L1 co-blockade, even in anti-PD-1-resistant tumor types where monotherapy is ineffective [146]. In a notable preclinical study, the combination of anti-PD-1 and anti-TIGIT therapy in mesothelioma animal models resulted in a 90% overall response rate, with complete tumor regression and long-term tumor-free survival surpassing 300 days, significantly exceeding the standard-of-care combination of anti-PD-1 and anti-CTLA-4, which yielded only a 60% response rate and induced more adverse effects [147].

### 7.2. Fc-Optimized Antibody Engineering

**Figure 16:**
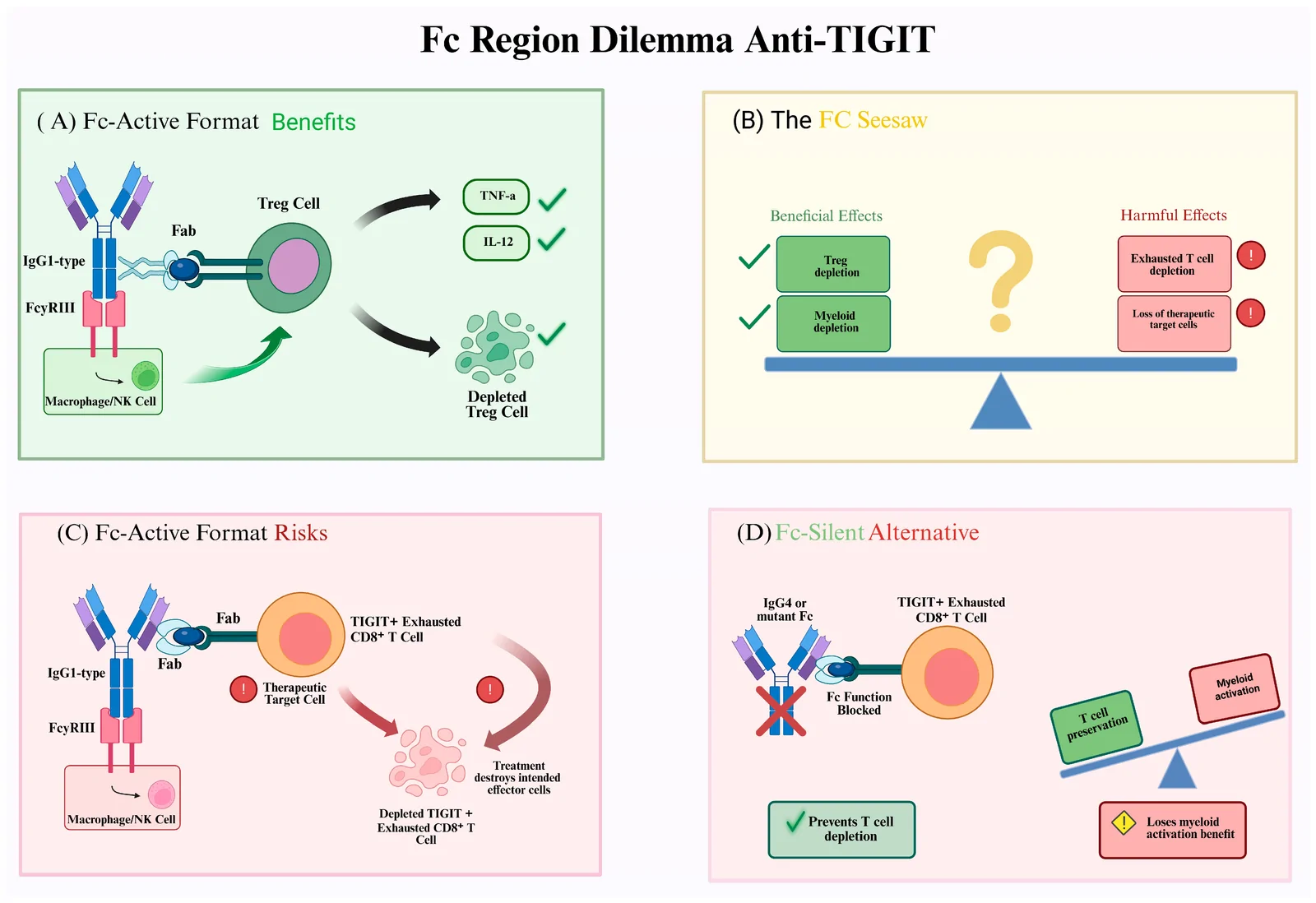
The Fc Region Dilemma in Anti-TIGIT Antibody Design. Four-panel figure illustrating the central pharmacological paradox of anti-TIGIT Fc engineering: (**A**) Fc-active (IgG1) benefits—FcγRIIIA engagement on macrophages/NK cells drives ADCC/ADCP-mediated Treg depletion and myeloid activation; (**B**) the Fc seesaw, balancing beneficial immune activation against the risk of depleting therapeutic target cells; (**C**) Fc-active risks, paradoxical ADCC-mediated elimination of TIGIT-expressing exhausted CD8^+^ TILs; (**D**) Fc-silent alternative, N297A/LALA/LALAPG mutant formats preserve exhausted T cells but sacrifice Treg depletion and myeloid activation benefits. Fc, fragment crystallizable region; TIGIT, T cell immunoreceptor with Ig and ITIM domains; FcγRIII, Fc gamma receptor III; NK cells, natural killer cells; Tregs, regulatory T cells; TNF-α, tumor necrosis factor-alpha; IL-12, interleukin-12; IgG, immunoglobulin G. Created in BioRender. Smail, S. (2026) https://BioRender.com/b4fanvf.

The comprehension of the dual function of the Fc region is propelling the development of a new generation of rationally engineered anti-TIGIT antibodies. Fc-active formats, exemplified by tiragolumab’s IgG1 scaffold, can effectively restructure the immunosuppressive TME by activating macrophages, monocytes, and dendritic cells via Fc gamma receptor interaction, while concurrently depleting Tregs and promoting CD8^+^ T cells towards a more functional memory-like phenotype (Figure 16) [40]. In contrast, Fc-silent formats may mitigate the risk of depleting exhausted effector T cells essential for anticancer immunity [83]. Belrestotug, a novel anti-TIGIT antibody assessed in two clinical trials for advanced solid tumors (2025), exhibited that its combination with anti-PD-1 therapy could diminish TIGIT-positive Tregs within tumors, enhance CD8 IFN-gamma (IFN-γ) expression, and restructure the TME, with spatial analysis indicating that inflamed and excluded immunotypes may act as predictive biomarkers of response as reported in a recent preprint [72].

The relative superiority of Fc-active versus Fc-silent anti-TIGIT antibody formats remains unresolved. Fc-active IgG1 antibodies may enhance antitumor immunity through FcγR-mediated engagement of macrophages and NK cells, promoting intratumoral Treg depletion, myeloid activation, and increased inflammatory cytokine production such as TNF-α and IL-12 as illustrated in Figure 16. However, the same Fc-dependent mechanism may also eliminate TIGIT-positive exhausted CD8^+^ T cells, potentially removing therapeutic target cells and weakening antitumor immunity. In contrast, Fc-silent antibodies such as domvanalimab may preserve TIGIT-positive effector T cells by blocking Fc-mediated depletion, although this may reduce the benefit of myeloid activation. Therefore, the Fc-region dilemma reflects a balance between beneficial Treg depletion and the risk of losing exhausted but potentially reinvigoratable antitumor T cells. Domvanalimab, developed by Arcus/Gilead as an Fc-silent IgG1 antibody, differs mechanistically from the Fc-active design of tiragolumab. In the randomized phase II ARC-10 trial involving patients with PD-L1-high NSCLC, domvanalimab demonstrated clinically meaningful improvements in ORR and PFS compared with zimberelimab monotherapy [113]. The NSCLC indication continues to be evaluated in the ongoing phase III STAR-121 trial [68]. However, the discontinuation of the parallel STAR-221 trial in gastric and esophageal cancer in December 2025—following a futility analysis demonstrating no improvement in OS—has significantly narrowed the scope of the domvanalimab program and dampened enthusiasm for TIGIT blockade in gastrointestinal malignancies [25].

Collectively, current evidence does not support a universally optimal Fc design for anti-TIGIT therapy. Fc-enabled antibodies offer the potential advantage of depleting TIGIT^+^ Tregs and remodeling suppressive myeloid compartments, effects that have been associated with enhanced antitumor immunity in both preclinical and translational studies [40,84]. However, because TIGIT is also expressed on exhausted but potentially reinvigoratable CD8^+^ T cells and NK cells, the same Fc-mediated effector mechanisms may inadvertently eliminate cellular populations required for therapeutic efficacy [83]. Conversely, Fc-silent antibodies preserve TIGIT-expressing effector lymphocytes but may sacrifice potentially beneficial Treg depletion and myeloid-cell activation [67,139]. Therefore, the central challenge may not be determining whether Fc-active or Fc-silent antibodies are universally superior, but rather identifying the specific Tumor and immune contexts in which each approach is most appropriate. This unresolved trade-off remains one of the most important unanswered questions in TIGIT immunotherapy and highlights the need for biomarker-driven selection strategies capable of defining when Treg depletion, effector-cell preservation, or a balance of both mechanisms is most likely to provide clinical benefit [40,67,83,139].

### 7.3. Bispecific Antibodies

Bispecific antibodies represent a next-generation therapeutic strategy with several mechanistic advantages over the administration of two separate monoclonal antibodies, particularly their ability to simultaneously engage tumor cells and immune cells in close physical proximity, as summarized in Table 6. Zhong et al. (2022) [148] created BiPT-23, a bispecific antibody of the IgG1 subclass that targets both PD-L1 and TIGIT. This antibody selectively eliminates PD-L1-positive tumor cells and TIGIT-positive Tregs while preserving CD11b+F4/80+ myeloid cells within the TME, thus averting the widespread immune cell depletion associated with certain monoclonal antibody therapies [148]. HB0036, another bispecific targeting PD-L1 and TIGIT, demonstrated the ability to simultaneously engage PD-L1-positive tumor cells and TIGIT-positive T cells, enhance CD226 expression, and elicit a superior T-cell proliferative response relative to the concurrent use of the parental antibodies. This resulted in enhanced tumor control in both syngeneic and xenograft models, facilitating its progression into a phase I clinical trial (NCT05417321) [149]. A 2026 study in Frontiers in Immunology further shown that tumor control attained via PD-L1 and TIGIT co-blockade using bispecific antibodies can be further augmented by incorporating vascular endothelial growth factor (VEGF) inhibition, thereby establishing a triple-targeting strategy [99]. The Fc-engineered bispecific YH41723 (IMC-202), which targets TIGIT and PD-L1, resulted in complete tumor regression in 7 out of 8 mice at the maximum dosage and elicited lasting immunologic memory upon tumor rechallenge, exhibiting enhanced efficacy compared to the combination of two monoclonal antibodies [150].

In addition to PD-L1 × TIGIT bispecifics, innovative bispecific formats aimed at other co-stimulatory and co-inhibitory pathways are emerging. ABL112, a pioneering bispecific antibody that targets both TIGIT and 4-1BB, was presented at the American Association for Cancer Research (AACR) 2024. The justification stems from the discovery that 4-1BB is increased on intratumoral Tregs; thus, dual targeting could concurrently deplete Tregs through TIGIT-dependent and 4-1BB-dependent pathways while activating effector T cells by TIGIT-dependent 4-1BB clustering. In murine tumor models, ABL112 exhibited enhanced efficacy relative to anti-TIGIT monoclonal antibodies, resulting in full tumor regression and sustained immunological memory following rechallenge [151]. AstraZeneca’s rilvegostomig, a PD-1 × TIGIT bispecific antibody with a unique molecular structure compared to the previously mentioned PD-L1 × TIGIT bispecifics, is currently undergoing active phase III clinical trials as of early 2026 and is one of only two prominent anti-TIGIT clinical initiatives still in progress [78,80].

### 7.4. TIGIT-Fc-LIGHT Bifunctional Fusion Proteins

Kyung et al. (2022) [73] in the Journal of Immunology developed TIGIT-Fc-LIGHT, a bifunctional fusion protein that associates TIGIT inhibition with TNFSF14 (LIGHT) costimulation, primarily aimed at resolving the issue of CD226 downregulation. LIGHT directly stimulates myeloid cells via the lymphotoxin beta receptor, independent of a functional Fc domain for Fc gamma receptor engagement, and coactivates CD8^+^ T and NK cells through herpes virus entry mediator (HVEM), which exhibits broader expression than CD226 on T memory stem cells and tumor-infiltrating lymphocytes across various tumor types. This is of paramount importance as it offers immunological costimulation that functions independently of both PD-1/PD-L1 suppression and CD226 expression, thereby expanding the clinical applicability of TIGIT blocking to PD-L1-low or checkpoint-resistant malignancies. The TIGIT-Fc-LIGHT construct exhibited significant antitumor efficacy in preclinical tumor models demonstrating both initial and acquired resistance to PD-1 inhibition [73].

### 7.5. Integration with CAR-T Cell Therapy

**Figure 17:**
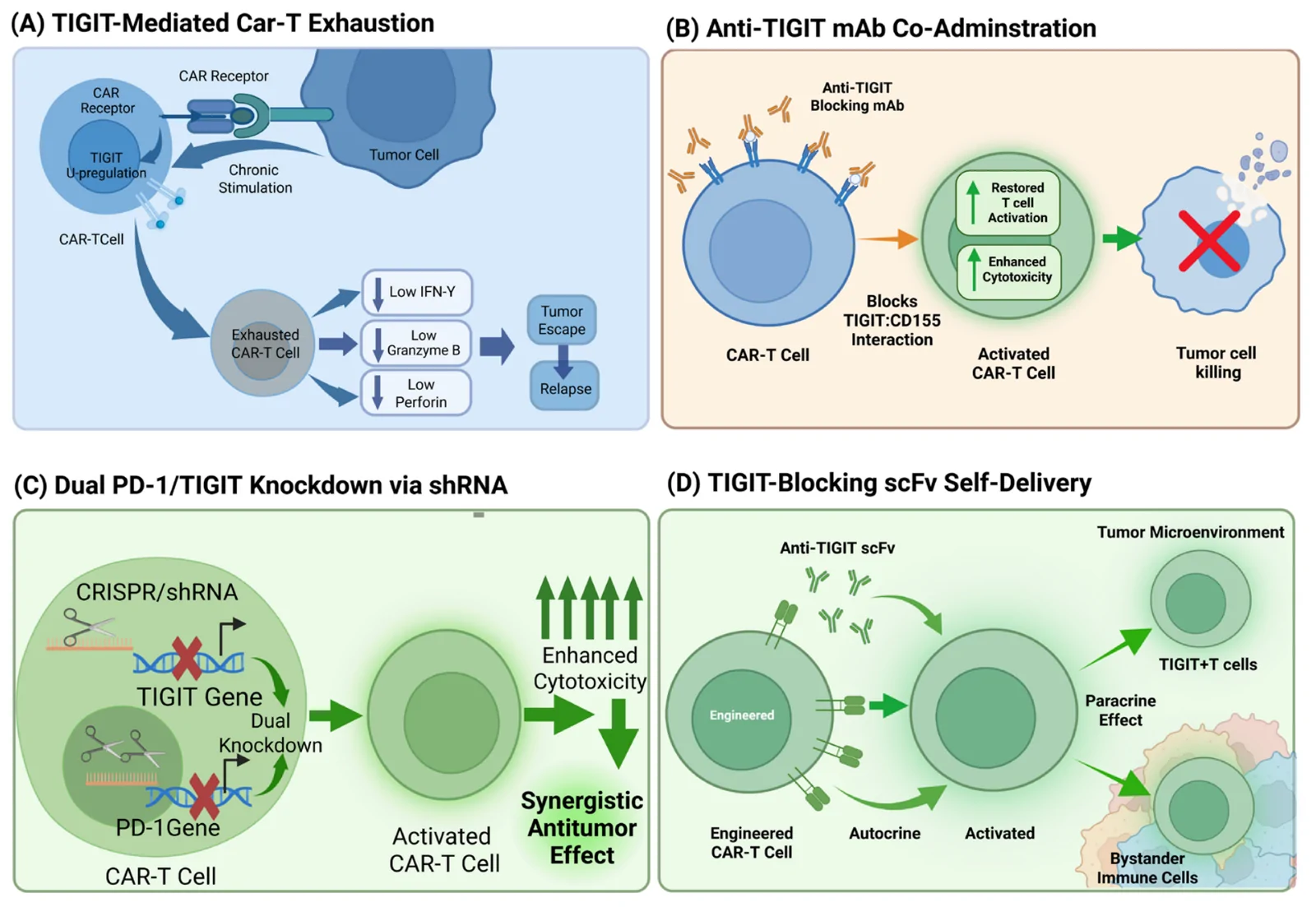
TIGIT in CAR-T Cell Therapy: Strategies to Overcome Exhaustion. Four-panel figure on CAR-T engineering: (**A**) TIGIT upregulation under chronic antigen stimulation drives CAR-T exhaustion and tumor relapse; (**B**) co-administration of anti-TIGIT mAb restores cytotoxic function; (**C**) dual PD-1/TIGIT shRNA knockdown provides cell-intrinsic, permanent checkpoint silencing with superior tumor control over single knockdown; (**D**) armored CAR-T cells secreting anti-TIGIT scFv provide both autocrine and paracrine checkpoint blockade within the TME, circumventing systemic toxicity. TIGIT, T cell immunoreceptor with Ig and ITIM domains; CAR-T, chimeric antigen receptor T-cell; IFN-γ, interferon-gamma; mAbs, monoclonal antibodies; PD-1, programmed cell death protein 1; CRISPR, clustered regularly interspaced short palindromic repeats; shRNA, short hairpin RNA; scFv, single-chain variable fragment. Green arrows indicate activation/enhancement of CAR-T-cell function. Red cross symbols indicate inhibition of exhaustion pathways or blockade of suppressive signaling. Created in BioRender. Smail, S. (2026) https://BioRender.com/gm3d3j9.

TIGIT has become a pivotal modulator of CAR-T cell depletion and therapeutic resistance in various cancer types. CAR-T cell therapy can be significantly impaired by TIGIT-mediated T-cell exhaustion, which develops following chronic antigen stimulation within the TME. Several therapeutic strategies have therefore been proposed to restore CAR-T functionality, including anti-TIGIT monoclonal antibody co-administration, dual PD-1/TIGIT gene knockdown, and engineered CAR-T cells capable of secreting TIGIT-blocking scFv molecules. These approaches enhance T-cell activation, cytotoxicity, cytokine production, and anti-tumor immune responses while reducing exhaustion-associated dysfunction. Finally, as shown in Figure 17, advanced TIGIT-targeting strategies may substantially improve CAR-T therapeutic efficacy and overcome tumor immune escape mechanisms.

Yuan et al. (2025) [153] demonstrated that T cells from multiple myeloma patients who relapsed early following B-cell maturation antigen (BCMA)-CAR-T therapy displayed elevated TIGIT expression compared to those with sustained responses. Furthermore, increased TIGIT levels were associated with greater tumor burden and unfavorable prognosis. Although TIGIT blockade had minimal impact on CAR-T function in vitro, it significantly promoted CAR-T proliferation, alleviated T cell exhaustion, and enhanced antitumor efficacy in vivo [153]. Lee et al. (2021) [154] published in Molecular Therapy a refined method utilizing a lentiviral two-in-one CAR-T system featuring a dual short-hairpin RNA cassette to concurrently downregulate two checkpoint receptors. Among the four combinations evaluated (PD-1/TIM-3, PD-1/LAG-3, PD-1/CTLA-4, and PD-1/TIGIT), the PD-1/TIGIT dual downregulation uniquely demonstrated synergistic antitumor effects, as PD-1 downregulation enhanced short-term effector function while TIGIT downregulation primarily maintained a less-differentiated, less-exhausted phenotype. This provided a mechanistic rationale for the observed synergy, culminating in a clinical trial (NCT04836507) [154]. Yang et al. (2023) employed an alternative strategy by genetically modifying anti-mesothelin (MSLN) CAR-T cells to continuously produce TIGIT-blocking single-chain variable fragments, illustrating that this self-delivery method improved CAR-T cell infiltration and activation within the TME, resulting in superior tumor regression in vivo [155]. Shen et al. (2024) recently introduced an innovative approach utilizing a mutated TIGIT co-receptor with improved CD155 binding affinity as a co-stimulatory signal in CAR-T cells, successfully transforming the inhibitory TIGIT-CD155 interaction into an activating signal that resulted in markedly elevated IFN-γ levels and improved antitumor efficacy in bladder cancer models [156].

### 7.6. Combination with Radiotherapy

Preclinical evidence indicates that the incorporation of TIGIT blocking with PD-1 inhibition and localized radiotherapy yields synergistic anticancer benefits, especially in immunologically “cold” cancers that generally exhibit resistance to checkpoint blockade alone. In a murine model of triple-negative breast cancer, the combination of anti-TIGIT, anti-PD-1, and localized radiotherapy resulted in the most significant growth delay of both irradiated primary tumors and unirradiated secondary tumors compared to all treatment groups. This combination also yielded the highest plasma concentrations of interferon-beta and interferon-gamma, a notable increase in CD8^+^ T cell infiltration, heightened CD226 expression on CD8^+^ T cells, and a reduction in Tregs within tumors, tumor-draining lymph nodes, and the spleen [98]. The incorporation of TIGIT blockade with anti-PD-1 and radiotherapy markedly diminished the quantity of metastatic lung nodules and promoted a less exhausted phenotype in CD8^+^ tumor-infiltrating lymphocytes, indicating that this triad may surmount the therapeutic resistance of ICIs in breast cancer and other immunologically inert tumor types [98].

### 7.7. Triple Pathway Blockade (TIGIT + PD-L1 + TGF-Beta)

Simultaneously addressing three distinct immunosuppressive pathways is an alternative option to surmount the duplication of suppressive mechanisms that constrains single or dual checkpoint blocking. Preclinical studies have shown that immune targeting of three distinct suppressive pathways TIGIT/CD155, PD-1/PD-L1, and TGF-β yields substantial antitumor efficacy in immune checkpoint-resistant models. This supports the hypothesis that strategically designed combination therapies addressing multiple non-overlapping immunosuppressive mechanisms are necessary to attain durable responses and avert the emergence of treatment-refractory disease [97].

### 7.8. TME Remodeling for “Cold” Tumors

In immunologically cold or immune-excluded cancers, less TIGIT expression at the bulk tissue level typically indicates restricted lymphocyte infiltration into the tumor rather than a lack of a therapeutic target, implying that a sequential strategy of “remodel first, release second” may be required. This method entails initially normalizing tumor vasculature, alleviating stromal and chemokine barriers, or inducing type I and type III interferon signaling to prepare the TME and enhance lymphocyte infiltration, subsequently implementing dual TIGIT and PD-1 checkpoint blockade after immune cells have infiltrated the tumor. In cancers exhibiting lack of MHC class I, which redirects immune surveillance towards innate immune cells, TIGIT inhibition should be integrated with NK cell-enhancing strategies rather than depending exclusively on T cell-mediated mechanisms [139].

A different strategy for tackling the cold tumor issue entails localized TIGIT blockage instead of systemic delivery. Um et al. (2025) [152] delineated a bispecific antibody, chi2B5 × 4F11, targeting TIGIT and CDCP1, particularly engineered for pancreatic ductal adenocarcinoma. This antibody facilitates TIGIT inhibition inside CUB domain-containing protein 1 (CDCP1)-positive TMEs, hence promoting localized immune activation in the TME. This method improved NK cell-mediated cytotoxicity and stimulated pro-inflammatory cytokine release in vitro, while in humanized mouse models, it decreased the percentage of TIGIT-positive circulating immune subsets within the CD226-positive compartment, indicating a functional restoration of co-stimulatory signaling via tumor-targeted rather than systemic TIGIT inhibition [152].

### 7.9. Predictive Biomarker Development and Precision Patient Selection for Anti-TIGIT Immunotherapy

A major factor contributing to the inconsistent clinical efficacy of anti-TIGIT immunotherapy is the absence of robust predictive biomarkers capable of identifying patients most likely to benefit from treatment. Unlike PD-1/PD-L1 blockade, where biomarker-guided patient selection is routinely incorporated into clinical practice, anti-TIGIT development has largely proceeded without validated enrichment strategies. This limitation is particularly important because TIGIT biology is highly context-dependent and influenced by ligand availability, co-inhibitory receptor networks, immune cell composition, and the mechanism of action of individual anti-TIGIT antibodies. Consequently, accumulating evidence indicates that TIGIT expression alone is insufficient as a predictive biomarker and that effective patient selection requires evaluation of the broader TIGIT signaling axis and the surrounding TME [9,40].

Recent translational studies demonstrate that TIGIT should be interpreted within a broader checkpoint network. TIGIT is commonly co-expressed with PD-1, LAG-3, and TIM-3 on exhausted T cells and NK cells, while also being highly expressed on suppressive intratumoral Tregs. Multi-omics analyses indicate that these co-expression patterns are more informative than TIGIT expression alone and more accurately reflect the immune state associated with response or resistance to immunotherapy [125].

A conceptual limitation of many current biomarker strategies is their reliance on quantification of a single checkpoint receptor. However, the immunological synapse operates as an integrated signaling network in which multiple co-inhibitory and co-stimulatory pathways collectively regulate T-cell activation, exhaustion, tolerance, and reinvigoration rather than functioning independently [157]. Within this framework, TIGIT expression alone provides limited biological information because its functional significance depends on the simultaneous status of competing co-stimulatory pathways, particularly CD226, CD28, and ICOS, as well as co-inhibitory receptors including PD-1, LAG-3, and TIM-3 [9,157]. Consequently, a TIGIT-high Tumor may remain responsive, non-responsive, or even resistant to TIGIT blockade depending on the net balance of activating and inhibitory signals present at the immune synapse. This concept is supported by our bioinformatics analyses, which demonstrated that broader checkpoint-balance signatures carry greater predictive value than isolated TIGIT measurements, and by recent transcriptomic studies showing that multi-checkpoint expression patterns better capture immunotherapy responsiveness than individual checkpoint markers alone [125]. Therefore, future biomarker development should move beyond TIGIT-centric models toward integrated immune-synapse profiling that captures the functional equilibrium between co-stimulatory and co-inhibitory networks within the TME [9,125,157].

Among currently proposed biomarkers, CD226 status is the most promising mechanistically. TIGIT suppresses antitumor immunity by competing with CD226 for binding to CD155. Progressive loss of CD226 on exhausted CD8^+^ TILs has been associated with immune dysfunction, whereas CD226-high CD8^+^ T cells remain responsive to anti-TIGIT therapy. Furthermore, a high TIGIT/CD226 ratio, particularly within Tregs, correlates with unfavorable outcomes following checkpoint blockade. These findings suggest that the balance between TIGIT and CD226 may be a more relevant predictor of benefit than TIGIT expression alone [9,116,131,132,133].

CD155 expression is another strong biomarker candidate. Since anti-TIGIT therapies target the TIGIT–CD155 axis, tumors with high CD155 expression may be more dependent on this pathway for immune evasion. Elevated CD155 has been associated with acquired resistance to immunotherapy, poor prognosis, and enhanced activity of combined TIGIT and PD-1 blockade. Importantly, prospective patient enrichment based on CD155 expression has not yet been implemented in phase III studies, representing a major opportunity for future clinical development [51,124,138,139].

Clinical evidence also highlights the importance of the overall immune microenvironment. In the CITYSCAPE trial, the greatest benefit from tiragolumab plus atezolizumab was observed in PD-L1-selected tumors, establishing PD-L1 as the first practical enrichment biomarker for anti-TIGIT therapy [58]. Subsequent analyses identified high baseline TAM and Treg signatures as predictors of response, suggesting that immune-active but immunosuppressive TMEs may be particularly susceptible to TIGIT-targeted treatment (1). Similarly, biomarker analyses from AdvanTIG-105 showed that combining PD-L1 with TIGIT-related biomarkers, including TIGIT expression, CCR8, or TAM-associated signatures, improved patient stratification and identified subgroups with substantially prolonged PFS [126].Additional exploratory biomarkers include tumor immunotype classification (inflamed versus excluded), spatial immune cell distribution, circulating PD-1^+^TIGIT^+^CD8^+^ populations, and circulating tumor DNA (ctDNA) kinetics during therapy [72,158]. However, these approaches remain investigational and require prospective validation before routine clinical implementation.

Current evidence further suggests that patient selection should integrate both molecular biomarkers and tumor-specific biological context. The contrasting outcomes observed across anti-TIGIT phase III studies indicate that tumors are not equally dependent on the TIGIT–CD226–CD155 axis. For example, the positive results observed in ESCC compared with the negative outcomes in SCLC and HCC suggest that baseline immune inflammation, CD155 expression, checkpoint network architecture, and responsiveness to PD-L1 therapy may collectively determine the likelihood of benefit from TIGIT blockade [22,26,58,144]. Consequently, future anti-TIGIT trials should move beyond single-marker enrichment and adopt composite stratification strategies incorporating tumor histology, PD-L1 status, CD226 functionality, CD155 expression, and immune-context signatures to identify biologically relevant responder populations [9,40,124,126].

Current evidence supports a tiered biomarker strategy for anti-TIGIT immunotherapy (Table 7). Tier 1 consists of PD-L1 and tumor histology, which are currently clinically deployable [21,58]. Tier 2 includes CD226 status and CD155 expression, which have strong biological rationale and are ready for prospective validation [9,124,132]. Tier 3 comprises composite immune-context signatures incorporating multiplex IHC, spatial transcriptomics, single-cell profiling, TAM/Treg density, and circulating immune biomarkers [40,125,126]. Collectively, these findings indicate that future patient selection for anti-TIGIT therapy will likely depend on multimodal biomarker panels rather than TIGIT expression alone, potentially overcoming one of the major barriers to the clinical success of anti-TIGIT immunotherapy.

Despite substantial biological interest, no individual biomarker has yet demonstrated sufficient predictive performance to support routine clinical implementation of anti-TIGIT therapy. TIGIT expression itself appears to have limited clinical utility because it does not distinguish between biologically distinct TIGIT-positive populations, including dysfunctional effector T cells, NK cells, and highly suppressive Tregs. In contrast, CD226 status has stronger mechanistic validity because restoration of CD226-mediated signaling represents the principal therapeutic mechanism of TIGIT blockade. Similarly, CD155 expression may identify tumors that are functionally dependent on the TIGIT–CD155 axis but remains unvalidated as a prospective enrichment marker. Collectively, current evidence suggests that composite biomarker approaches integrating TIGIT-axis components with immune-context information are likely to outperform any single biomarker strategy. Future clinical development will therefore require multiplex and multi-omic approaches capable of simultaneously assessing checkpoint networks, immune cell composition, spatial organization, and co-stimulatory versus co-inhibitory balance within the TME.

## 8. Prioritization of Future Development Strategies

Importantly, not all proposed solutions currently possess the same level of supporting evidence or translational readiness. Based on available clinical and mechanistic data, biomarker-guided patient selection and rational combination strategies incorporating PD-1/PD-L1 blockade remain the most mature approaches for improving anti-TIGIT efficacy (Table 8) [19,40,58,68,113]. These strategies benefit from extensive clinical experience and directly address the two factors most consistently associated with anti-TIGIT failure: inadequate patient selection and checkpoint network redundancy.

A second tier of development includes Fc-optimized antibodies and bispecific checkpoint-targeting agents, which are supported by strong mechanistic rationale and encouraging preclinical and early clinical findings but still require confirmation in randomized clinical trials [78,145,146,148,160]. These approaches seek to overcome limitations of first-generation anti-TIGIT antibodies through improved immune-synapse modulation and more efficient targeting of suppressive cellular compartments.

By comparison, TIGIT-Fc-LIGHT fusion proteins, CAR-T engineering approaches, radiotherapy-based combinations, and triple-pathway blockade should currently be regarded as exploratory strategies. Although these approaches have demonstrated promising activity in preclinical studies, clinical evidence remains limited, and their ultimate therapeutic value is still uncertain [73,97,98,150,151,152,154]. Consequently, they should be viewed as hypothesis-generating platforms rather than near-term solutions for overcoming the limitations of current anti-TIGIT therapies.

Overall, the strongest evidence currently supports a precision immunotherapy framework combining biomarker-guided patient selection, preservation of CD226 signalling, and mechanism-based checkpoint combinations. More complex multifunctional platforms may ultimately prove beneficial but require substantially greater clinical validation before their role in routine cancer immunotherapy can be established.

## 9. Contribution of This Review

The rapid expansion of the TIGIT literature has resulted in numerous reviews describing TIGIT biology, checkpoint signaling, preclinical studies, and ongoing clinical development. However, most reviews were published during periods of optimism when anti-TIGIT therapy was still considered one of the most promising next-generation checkpoint strategies. Consequently, many focused primarily on biological rationale and therapeutic potential rather than on understanding the causes of clinical failure.

This review adopts a different perspective. Rather than asking why TIGIT emerged as an attractive immunotherapy target, we ask why the majority of anti-TIGIT clinical programs ultimately failed despite encouraging biological and early clinical signals. Particular emphasis is placed on lessons learned from negative phase III studies, including SKYSCRAPER-01, SKYSCRAPER-02, SKYSCRAPER-03, SKYSCRAPER-14/IMbrave152, STAR-221, KEYVIBE, and AdvanTIG trials, and on identifying the biological and translational factors most likely responsible for these outcomes.

Several features distinguish this review from previous publications. First, we include original bioinformatics analyses evaluating the TIGIT-CD226 axis across multiple cancer datasets and immunotherapy-treated cohorts, providing independent evidence that isolated *TIGIT* expression is insufficient for patient stratification and supporting a broader checkpoint network model. Second, we develop a precision immunotherapy framework centered on biomarker-guided patient selection, tumor-specific dependence on the TIGIT-CD155-CD226 axis, and rational combination strategies. Third, we critically rank proposed resistance mechanisms and future therapeutic solutions according to the strength of mechanistic, translational, and clinical evidence rather than presenting all hypotheses as equally supported.

Most importantly, this review argues that anti-TIGIT failure should not be interpreted as evidence that TIGIT is an invalid immunotherapy target. Instead, current data support a more nuanced model in which clinical outcomes are determined by the interaction of biomarker selection, CD226 functionality, tumor-specific biology, checkpoint network architecture, Fc-dependent effects, and trial design. Consequently, the future of TIGIT-based therapy is likely to depend less on broader application of checkpoint blockade and more on precision immunotherapy strategies that identify the specific patients, Tumor types, and biological contexts in which TIGIT inhibition remains therapeutically relevant.

## 10. Conclusions

TIGIT remains a biologically relevant immune checkpoint, but the largely negative outcomes of phase III trials demonstrate that biological rationale alone is insufficient for clinical success. Current evidence suggests that anti-TIGIT failure is not primarily due to invalid target biology, but rather reflects a combination of inadequate biomarker-guided patient selection, tumor-specific dependence on the TIGIT-CD155-CD226 axis, progressive CD226 loss in exhausted lymphocytes, checkpoint network redundancy, and trial-design limitations. A key lesson from the TIGIT field is that *TIGIT* expression alone is an inadequate biomarker. Instead, therapeutic responsiveness is more likely determined by the broader balance of co-stimulatory and co-inhibitory signals operating at the immune synapse, particularly the functional status of *CD226*, *CD155* expression, and the surrounding immune context. Future biomarker strategies should therefore move beyond single-marker approaches toward integrated, multi-parameter immune profiling.

The future of TIGIT-directed therapy lies in precision immunotherapy rather than broad checkpoint blockade. Biomarker-guided patient selection, identification of TIGIT-dependent tumor types, preservation of CD226 signaling, and rational mechanism-based combination strategies represent the most scientifically supported paths forward. Thus, the principal contribution of this review is to reframe the TIGIT story not as the failure of a target, but as a lesson in the importance of biological context, patient stratification, and precision-guided immunotherapy development.

## Figures and Tables

**Table 1:** Major CD155/CD226/TIGIT axis Family Ligands and Their Interactions with TIGIT-Axis Immune Checkpoint Receptors in Tumor Immunity.

Ligand	Receptor(s)	Type of Signal	Cellular Expression (Ligand)	Biological Effect	Key Evidence (PubMed/Scopus)
CD155	TIGIT	Inhibitory	Tumor cells, APCs	Suppresses T-cell and NK-cell activation; promotes immune evasion	TIGIT binds CD155 with high affinity and inhibits immune responses [4,28]
	CD226	Activating	Tumor cells, APCs	Enhances cytotoxic T-cell and NK-cell activity	CD226 competes with TIGIT for CD155 binding, delivering activation signals [4,19]
	CD96	Mostly inhibitory (context-dependent)	Tumor cells, APCs	Regulates NK-cell function; may suppress anti-tumor immunity	CD96 shares CD155 ligand and modulates immune responses [29]
CD112	TIGIT	Inhibitory	Tumor cells, APCs	Weak inhibitory signaling compared to CD155	TIGIT binds CD112 with lower affinity [30]
	CD226	Activating	Tumor cells, APCs	Promotes T-cell and NK-cell cytotoxicity	CD226–CD112 interaction supports immune activation [31]
	CD112R	Inhibitory	Tumor cells, APCs	Suppresses T-cell activation and proliferation	CD112R delivers inhibitory signals via CD112 binding [32]
CD113	TIGIT	Inhibitory (less characterized)	Tumor cells, APCs	Potential contribution to immune suppression	Identified as additional TIGIT ligand in multiple studies [33]
Nectin-4	TIGIT	Inhibitory (emerging)	Tumor cells (e.g., epithelial cancers)	Possible tumor-specific immune evasion mechanism	Evidence suggests TIGIT–nectin-4 interaction (limited functional data) [34]
CD111	CD96/CD226 (indirect axis)	Activating/Regulatory	APCs, tumor cells	Contributes to broader nectin network signaling	Part of CD155/CD226/TIGIT ligand family interacting with checkpoint receptors [35]

This table summarizes the major CD155/CD226/TIGIT family ligands and their interactions with TIGIT-axis receptors involved in anti-tumor immune regulation. CD155 and CD112 are the best-characterized ligands, whereas CD113, Nectin-4, and CD111 are less established or context-dependent. Abbreviations: APCs, antigen-presenting cells; CD, cluster of differentiation; TIGIT, T-cell immunoreceptor with Ig and ITIM domains; NK cells, natural killer cells.

**Table 2:** Pan-Cancer Expression Patterns and Prognostic Significance of TIGIT in Major Human Cancers.

Cancer Type	TIGIT Expression Level	Key Expression Details	Prognostic Impact	Key Data Sources
NSCLC (Lung)	High	Highest median expression rank (~60/61 out of 100) across pan-cancer cohorts; 36% of lung cancers classified as TIGIT-high; TIGIT co-expressed with PD-1, PD-L1, TIM3.	TIGIT-high NSCLC patients treated with pembrolizumab had significantly better OS (44 vs. 23 months) and ORR (60% vs. 39%).	[46] (*n* = 15,630 pan-cancer); [47] (*n* = 24,186)
Head & Neck SCC	High	Median rank ~59; high frequency of TIGIT^+^ lymphocytes by IHC, particularly in squamous cell histology.	Favorable prognosis with high TIGIT in HNSC (TCGA).	[47,48]
Cervical Cancer	High	Median rank ~55 in large cohort.	Data limited.	[47]
Melanoma (SKCM)	High	TIGIT upregulated on TILs; co-expressed with PD-1 on CD8^+^ T cells. DNA methylation regulates TIGIT expression in the melanoma TME.	Favorable prognosis in SKCM (TCGA pan-cancer analysis).	[49]
Colorectal Cancer	Moderate/High	18% TIGIT-high in clinical cohort; TIGIT^+^ lymphocytes detected across all 86 tumor entities by IHC. High co-expression of CD155 and TIGIT predicts poor prognosis.	High CD155 + TIGIT co-expression = independent poor prognostic factor in CRC.	[50,51]
Gastric/Stomach Cancer	Moderate/High	20% TIGIT-high; strong co-expression with immunostimulators, immunoinhibitors, chemokines, and MHC molecules, especially in gastroesophageal cancer.	High TIGIT associated with worse OS in East Asian meta-analysis.	[50,52]
Breast Cancer	Moderate	20% TIGIT-high; CD155 expression associated with poor outcomes and more aggressive subtypes (HER2+, TNBC). TIGIT related to aggressiveness.	Favorable prognosis in BRCA (TCGA). Blocking TIGIT-CD155 enhanced immune-mediated lysis in vitro.	[50,53]
Pancreatic Cancer	Moderate/High	31% TIGIT-high (highest proportion in one clinical cohort); CD155 expression high (~50% CD155-high) by IHC. TIGIT delineates functionally distinct T-cell populations.	In pancreatic ductal adenocarcinoma, high intratumoral PD-1^+^TIGIT^−^ conventional T-cell levels were associated with improved clinical outcomes, whereas TIGIT expression marked more anti-inflammatory/exhausted T-cell phenotypes.	[50,54]

NSCLC, non-small cell lung cancer; SCC, squamous cell carcinoma; HNSC, head and neck squamous cell carcinoma; SKCM, skin cutaneous melanoma; CRC, colorectal cancer; BRCA, breast cancer; HER2+, human epidermal growth factor receptor 2-positive; TNBC, triple-negative breast cancer; PD-1, programmed cell death protein 1; PD-L1, programmed death-ligand 1; TIM3, T-cell immunoglobulin and mucin-domain containing-3; TIGIT, T-cell immunoreceptor with Ig and ITIM domains; CD, cluster of differentiation; TILs, tumor-infiltrating lymphocytes; IHC, immunohistochemistry; TME, tumor microenvironment; MHC, major histocompatibility complex; OS, overall survival; ORR, objective response rate; TCGA, The Cancer Genome Atlas.

**Table 3:** Anti-TIGIT Monoclonal Antibodies in Clinical Development.

Agent	Company	Antibody Format/Fc Design	Mechanism of Action	Key Clinical Trial(s)	ORR (Combination)	Common Adverse Events	Grade ≥ 3 AE Rate	Current Status	Refs
Tiragolumab	Roche/Genentech	Humanized IgG1/kappa, Fc-active	Blocks TIGIT-CD155; FcγR-mediated myeloid activation, Treg modulation, and CD8^+^ T-cell reprogramming from exhausted to memory-like state	Phase Ia/Ib (NCT03563716); CITYSCAPE (Phase II); SKYSCRAPER program (Phase III)	37% with atezolizumab in PD-L1-high (≥50%) NSCLC vs. 21% with atezolizumab alone (CITYSCAPE PD-L1-high subgroup); 31.3% vs. 16.2% in the overall PD-L1-selected population; 50% in NSCLC expansion cohort	Fatigue, anemia, rash, pruritus	4% in Phase I mono/combo	Withdrawn from pipeline (2025); most Phase III trials failed	[40,58,59,60]
Vibostolimab (MK-7684)	Merck/MSD	Humanized IgG1	Blocks TIGIT-CD155 and TIGIT-CD112 interactions	Phase I (NCT02964013); KeyVibe-003 (Phase III, NCT04738487)	31% with pembrolizumab in CPI-naive NSCLC with PD-L1 ≥ 1%; 7% monotherapy and 5% combo in CPI-refractory NSCLC	Pruritus, fatigue, rash, arthralgia, decreased appetite; grade 3–4 lipase increase and hypertension; one treatment-related death (pneumonitis)	Grade 3–4 TRAEs in 10/79 patients in refractory NSCLC cohort	Terminated (2025); Phase III negative	[61,62,65]
Ociperlimab (BGB-A1217)	BeiGene	Humanized IgG1, Fc-competent; C1q and FcγR binding; ADCC-capable	High-affinity TIGIT blockade; induces ADCC; synergistic immune activation with tislelizumab	AdvanTIG-105 (Phase I, NCT04047862); AdvanTIG-302 (Phase III, NCT04746924)	10% with tislelizumab in mixed solid tumors; DCR 50%	Fatigue, diarrhea; grade 3 immune-related AEs included colitis and low cortisol	62.5% grade ≥ 3 TEAEs; 50% serious TEAEs	Terminated; Phase III negative	[66]
Domvanalimab (AB154)	Arcus Biosciences/Gilead	Humanized IgG1, Fc-silent	Blocks TIGIT-CD155 without Treg depletion; enhances exhausted CD8^+^ T-cell activation through a lymph node-dependent mechanism	Phase II basket trial (NCT05724563); Phase III trials ongoing	17.2% with zimberelimab in anti-PD-1-refractory HCC	TRAEs in 55.2%, mostly low grade	Grade ≥ 3 TRAEs in 10.3%; SAEs in 6.9%	Phase II/III ongoing	[67]
Etigilimab (MPH313)	Mereo BioPharma	Humanized IgG1, FcγR-competent	Blocks TIGIT-CD155; reduces Tregs and TIGIT^+^ Tregs; increases CD8:Treg ratio; enhances NK-cell and effector memory T-cell activation	Phase Ia/b; ACTIVATE Phase Ib/II (NCT04761198)	25% ORR with nivolumab in ACTIVATE (3 CR + 7 PR/40 evaluable)	Rash, nausea, fatigue; in combination: decreased appetite, nausea, rash	6/33 patients grade ≥ 3 in Phase I; only 8/76 > grade 2 and 1 TRSAE in ACTIVATE	Phase Ib/II ongoing	[20,69,70]
EOS-448 (GSK4428859A)	iTeos Therapeutics/GSK	Fully human IgG1, Fc-active; picomolar FcγR engagement	Triple mechanism: TIGIT blockade, FcγR-mediated APC/myeloid modulation, and preferential depletion of TIGIT^+^ Tregs and terminally exhausted TIGIT-high CD8^+^ T cells while sparing effector T cells and Tpex	Phase I first-in-human; TIG-007 Phase I/II in RRMM	Early signs of efficacy	Good tolerability; depletion of TIGIT^+^ Tregs confirmed pharmacodynamically	Not yet fully reported	Phase I/II ongoing	[73,74]
COM902	Compugen	Fully human IgG4, Fc-silent/minimal effector function	Pure TIGIT-CD155 blockade without Fc-mediated effects	Phase I (NCT04354246)	No objective responses reported in dose-escalation monotherapy	Fatigue, diarrhea	2 DLTs: grade 2 nausea and grade 3 atrial fibrillation	Phase I; expansion with COM701 planned	[75]
M6223	EMD Serono/Merck KGaA	Fully human IgG1, Fc-active	Blocks TIGIT-CD155, TIGIT-CD112, and TIGIT-CD226; triple mechanism including direct blockade, CD226 activation, and Fc-mediated depletion of TIGIT^+^ subsets	Phase I first-in-human (NCT04457778), monotherapy ± bintrafusp alfa	Clinical benefit in 9/24 with monotherapy; 2/17 with bintrafusp alfa combination	TIGIT^+^ Treg depletion confirmed; adrenal insufficiency and anemia as DLTs	33% grade ≥ 3 with monotherapy; 71% grade ≥ 3 with bintrafusp alfa	Phase I ongoing	[76,77]

ADCC, antibody-dependent cellular cytotoxicity; AE, adverse event; APC, antigen-presenting cell; CD, cluster of differentiation; C1q, complement component 1q; CPI, checkpoint inhibitor; CR, complete response; DCR, disease control rate; DLT, dose-limiting toxicity; Fc, fragment crystallizable region; FcγR, Fc gamma receptor; HCC, hepatocellular carcinoma; IgG, immunoglobulin G; NK, natural killer; NCT, National Clinical Trial identifier; NSCLC, non-small cell lung cancer; ORR, objective response rate; PD-1, programmed cell death protein 1; Phase I/II/III, clinical trial phases I/II/III; PR, partial response; RRMM, relapsed/refractory multiple myeloma; SAE, serious adverse event; TEAE, treatment-emergent adverse event; TIGIT, T-cell immunoreceptor with immunoglobulin and immunoreceptor tyrosine-based inhibitory motif domains; Tpex, progenitor exhausted T cell; Treg, regulatory T cell; TRAE, treatment-related adverse event.

**Table 6:** Next-Generation Bispecific Anti-TIGIT Agents.

Agent	Company	Format	Targets	Key Trial	Preliminary Efficacy	Safety Profile	Refs
Rilvegostomig (AZD2936)	AstraZeneca	Monovalent bispecific humanized IgG1	PD-1 + TIGIT	ARTEMIDE-01 Phase I/II (NCT04995523); 2 Phase III trials (NCT06109779, NCT06357533)	4 confirmed PR and 33 SD in 83 CPI-pretreated NSCLC patients; 6-month DCR 31.3%; RP2D 750 mg	Well tolerated in CPI-pretreated patients	[80]
HB0036	Huaota Biopharmaceutical	Bispecific antibody	PD-L1 + TIGIT	Preclinical/early clinical	Greater T-cell proliferation than parental antibody combination in vitro; CD226 upregulation, PD-1 downregulation; improved tumor control in syngeneic and xenograft models	Preclinical stage	[99]
BiPT-23	Zhong et al. (academic)	IgG1 subclass bispecific antibody	PD-L1 + TIGIT	Preclinical only	Selectively eliminates PD-L1-positive tumor cells and TIGIT-positive Tregs while preserving CD11b+F4/80+ myeloid cells in the TME; avoids widespread immune cell depletion associated with monoclonal antibody monotherapy	Preclinical stage	[148]
YH41723 (IMC-202)	Innovent Biologics/IMC	Fc-engineered bispecific antibody	TIGIT + PD-L1	Preclinical; presented at AACR 2024	Complete tumor regression in 7/8 mice at maximum dose; durable immunologic memory upon tumor rechallenge; superior efficacy compared to combination of two monoclonal antibodies	Preclinical stage	[150]
ABL112	ABL Bio	Bispecific antibody	TIGIT + 4-1BB	Preclinical; presented at AACR 2024	Enhanced efficacy relative to anti-TIGIT monoclonal antibodies in murine tumor models; complete tumor regression and sustained immunological memory after rechallenge; dual Treg depletion via TIGIT-dependent and 4-1BB-dependent pathways	Preclinical stage	[151]
chi2B5 × 4F11	Um et al. (academic)	Bispecific antibody	TIGIT + CDCP1	Preclinical; pancreatic ductal adenocarcinoma-specific design	Improved NK cell-mediated cytotoxicity and pro-inflammatory cytokine release in vitro; decreased TIGIT-positive circulating immune subsets within the CD226-positive compartment in humanized mouse models	Preclinical stage	[152]

PR, partial response; SD, stable disease; DCR, disease control rate; RP2D, recommended phase 2 dose; CPI, checkpoint inhibitor; NSCLC, non-small cell lung cancer; PD-1, programmed cell death protein 1; PD-L1, programmed death-ligand 1; TIGIT, T-cell immunoreceptor with Ig and ITIM domains; 4-1BB, tumor necrosis factor receptor superfamily member 9; CDCP1, CUB domain-containing protein 1; TME, tumor microenvironment; Tregs, regulatory T cells; NK cells, natural killer cells; IgG1, immunoglobulin G subclass 1; Fc, fragment crystallizable region; AACR, American Association for Cancer Research; NCT, ClinicalTrials.gov identifier.

**Table 7:** Candidate Predictive Biomarkers for Anti-TIGIT Therapy: Evidence Level and Assay Readiness.

Biomarker	Mechanistic Rationale	Best Available Clinical Evidence	Assay Platform	Assay Readiness	Evidence Tier	Key Refs
PD-L1 (TPS/CPS)	Marker of pre-existing anti-tumor immunity; correlates with response to PD-(L)1 backbone	CITYSCAPE showed larger benefit in PD-L1-high [21]; SKYSCRAPER-01 enriched on PD-L1 [22]	IHC (22C3, SP263)	Deployable now (companion diagnostic)	Tier 1—necessary but not sufficient	[21,58]
TIGIT IHC alone	Direct target quantification	Retrospective TIGIT IHC did not separate responders from non-responders in CITYSCAPE or SKYSCRAPER	IHC (variable clones)	Available but unstandardised	Insufficient as single marker	[109,115]
CD226 status on CD8^+^ TIL	Anti-TIGIT works only when CD226 is present and phosphorylatable; CD226^hi^ CD8^+^ TIL required	CD226^hi^ prerequisite in mouse and human melanoma; pretreatment CD226^+^CD8^+^ correlates with response	Multiplex IF or flow cytometry	Available; needs cutoff standardisation	Tier 2—highest-priority to validate	[109,130,131,133]
Tumor CD155 IHC	Ligand engaged by anti-TIGIT; anti-TIGIT enhances anti-PD-1 specifically vs. CD155^hi^ Tumors	CD155^hi^ predicts poor anti-PD-1 monotherapy; CD155/TIGIT co-expression prognostic in CRC	IHC	Available; needs prospective cutoff	Tier 2	[51,124,138]
TIGIT/CD226 ratio on intraTumoral Tregs	High ratio marks Treg-dominant, therapy-resistant Tumors	Melanoma post-PD-1: high ratio predicts unfavourable outcome	Multiplex IF	Available; needs standardisation	Tier 2	[116,136]
CD155/PD-L1 ratio	Ratio determines which arm of a dual-blockade agent drives cytotoxicity	Direct demonstration with rilvegostomig murine surrogate	Multiplex IF	Preclinical to early clinical	Tier 2–3	[78]
CITYSCAPE macrophage/Treg gene signature	Post-hoc: baseline TAM + Treg density predicts tiragolumab benefit but not atezolizumab	Retrospective in phase II/III	Bulk RNA-seq deconvolution or multiplex IF	Available; needs prospective validation	Tier 3	[40]
Circulating PD-1^+^TIGIT^+^CD8^+^ frequency	On-treatment pharmacodynamic marker; predicts anti-PD-1 response	Melanoma/Merkel cell: post-treatment DPOS frequency correlates with clinical response and OS	Multi-parameter flow cytometry	Available; assay standardisation needed	Tier 3 (on-treatment PD marker)	[159]
Immune-synapse co-stimulatory/co-inhibitory balance signature	Composite readout of net synaptic input; captures TIGIT–CD226 balance and network state	Framework only; not yet validated	Multiplex IF + spatial transcriptomics	Hypothesis-generating	Tier 3	[127]
CD155 expression as cold-Tumor reprogramming marker	Pan-cancer computational rationale for CD155-guided enrichment in excluded/desert subset	Pan-cancer analysis only	Bulk RNA/IHC	Preclinical to hypothesis-generating	Tier 3	[139]

Evidence tiers reflect the maturity of biomarker validation for anti-TIGIT therapy: Tier 1 = clinically deployable; Tier 2 = validation-ready and requiring prospective confirmation; Tier 3 = exploratory or hypothesis-generating and requiring further assay standardization and clinical validation. “Necessary but not sufficient” indicates association with treatment benefit without adequate standalone predictive value, whereas “insufficient as a single marker” indicates lack of reliable predictive performance when used alone. PD-L1, programmed death-ligand 1; TPS, tumor proportion score; CPS, combined positive score; PD-(L)1, programmed death-1/programmed death-ligand 1; TIGIT, T-cell immunoreceptor with immunoglobulin and immunoreceptor tyrosine-based inhibitory motif domains; IHC, immunohistochemistry; CD, cluster of differentiation; TIL, tumor-infiltrating lymphocyte; IF, immunofluorescence; Treg, regulatory T cell; CRC, colorectal cancer; TAM, tumor-associated macrophage; RNA-seq, RNA sequencing; OS, overall survival; PD, pharmacodynamic.

**Table 8:** Prioritization of Future Anti-TIGIT Development Strategies According to Evidence Strength and Translational Readiness.

Strategy	Mechanistic Rationale	Highest Level of Evidence	Translational Readiness	Evidence Level	Key References
Biomarker-guided patient selection (PD-L1, CD226, CD155, immune signatures)	Addresses biological heterogeneity and identifies TIGIT-dependent tumors	Clinical biomarker analyses from CITYSCAPE, AdvanTIG-105, translational studies	Immediately implementable in future trials	Highest	[40,58,124,153,156]
PD-1/PD-L1 + TIGIT co-blockade	Restores CD226 signaling and addresses checkpoint redundancy	Multiple phase II/III clinical studies; one positive phase III study (SKYSCRAPER-08)	Clinically established platform	Highest	[19,26,58,68,113]
Fc-optimized anti-TIGIT antibodies	Balances Treg depletion, myeloid activation, and effector-cell preservation	Strong mechanistic and translational data; early clinical evidence	Clinical validation ongoing	High	[40,67,83,84,85,150,151]
Bispecific antibodies (PD-L1 × TIGIT, PD-1 × TIGIT, TIGIT × 4-1BB)	Simultaneous modulation of multiple immune-synapse pathways	Extensive preclinical evidence; early clinical activity	Active clinical development	Moderate–High	[78,80,85,148,150,151]
TIGIT-Fc-LIGHT fusion proteins	Bypasses CD226 dependence and enhances alternative costimulation	Preclinical efficacy only	Experimental	Moderate	[73]
Radiotherapy + TIGIT blockade	Converts cold tumors into inflamed tumors and improves T-cell infiltration	Strong preclinical evidence	Early translational stage	Moderate	[98]
Triple-pathway blockade (TIGIT + PD-L1 + TGF-β)	Simultaneously targets multiple resistance mechanisms	Preclinical validation only	Experimental	Moderate–Low	[97]
CAR-T integration and TIGIT-engineered cell therapies	Prevents TIGIT-mediated cellular exhaustion and improves persistence	Predominantly preclinical evidence	Early-stage development	Low	[153,154,155]

4-1BB, tumor necrosis factor receptor superfamily member 9 (CD137); CAR-T, chimeric antigen receptor T-cell; CD, cluster of differentiation; Fc, fragment crystallizable region; LIGHT, homologous to lymphotoxins, exhibits inducible expression, and competes with herpes simplex virus glycoprotein D for herpesvirus entry mediator, a receptor expressed by T lymphocytes (TNFSF14); PD-1, programmed cell death protein 1; PD-L1, programmed death-ligand 1; TGF-β, transforming growth factor-beta; TIGIT, T-cell immunoreceptor with immunoglobulin and immunoreceptor tyrosine-based inhibitory motif domains; Treg, regulatory T cell.

## Data Availability

No new data were created or analyzed in this study. Data sharing is not applicable to this article.
